# Inhalation and dermal exposure to biocidal products during foam and spray applications

**DOI:** 10.1093/annweh/wxad037

**Published:** 2023-07-08

**Authors:** Anja Schäferhenrich, Katharina Blümlein, Wolfgang Koch, Stefan Hahn, Katharina Schwarz, Ulrich Poppek, Ralph Hebisch, Urs Schlüter, Monika Krug, Thomas Göen

**Affiliations:** Institute and Outpatient Clinic of Occupational, Social, and Environmental Medicine, Friedrich-Alexander-Universität Erlangen-Nürnberg, Erlangen, Germany; Fraunhofer Institute for Toxicology and Experimental Medicine (ITEM), Hanover, Germany; Fraunhofer Institute for Toxicology and Experimental Medicine (ITEM), Hanover, Germany; Fraunhofer Institute for Toxicology and Experimental Medicine (ITEM), Hanover, Germany; Fraunhofer Institute for Toxicology and Experimental Medicine (ITEM), Hanover, Germany; Federal Institute for Occupational Safety and Health (BAuA), Dortmund, Germany; Federal Institute for Occupational Safety and Health (BAuA), Dortmund, Germany; Federal Institute for Occupational Safety and Health (BAuA), Dortmund, Germany; Federal Institute for Occupational Safety and Health (BAuA), Dortmund, Germany; Institute and Outpatient Clinic of Occupational, Social, and Environmental Medicine, Friedrich-Alexander-Universität Erlangen-Nürnberg, Erlangen, Germany

**Keywords:** occupational exposure, workplace measurements, air monitoring, biocides, pyrethroids, quaternary ammonium compounds

## Abstract

**Objectives:**

Foaming and spraying are common application techniques for biocidal products. In the past, inhalation and dermal exposure during spraying have been investigated extensively. Currently, however, no exposure data are available for foaming, hindering a reliable risk assessment for foam applications of biocidal products. The focus of this project was the quantification of inhalation and potential dermal exposure to non-volatile active substances during the foam application of biocidal products in occupational settings. In some settings, exposure during spray application was measured for comparative purposes.

**Methods:**

The inhalation and dermal exposure of operators were investigated during the application of benzalkonium chlorides and pyrethroids by foaming and spraying, considering both small- and large-scale application devices. Inhalation exposure was measured by personal air sampling; potential dermal exposure was measured using coveralls and gloves.

**Results:**

Potential dermal exposure was substantially higher than inhalation exposure. Changing from spraying to foaming reduced inhalation exposure to airborne non-volatile active substances, but had no relevant effect on potential dermal exposure. However, for potential dermal exposure, considerable differences were observed between the application device categories.

**Conclusions:**

To our knowledge, this study presents the first comparative exposure data for the foam and spray application of biocidal products in occupational settings with detailed contextual information. The results indicate a reduction of inhalation exposure with foam application compared to spray application. However, special attention is necessary for dermal exposure, which is not reduced by this intervention.

**What’s Important About This Paper?**

This paper presents the first comparative dermal and inhalation exposure data for the foam and spray application of biocidal products in occupational settings with detailed contextual information. Dermal exposures were higher than inhalation exposures, and the latter were reduced by changing from spraying to foaming applications. These data may be useful at both national and European levels for the authorisation and regulation of biocidal products for foam application.

## Introduction

In the course of authorising biocidal products (European Commission 2012), one aspect requiring careful consideration is occupational health and safety during the application of these products. The associated risk assessment is based on reference values for toxicological effects as well as on exposure for a given application scenario, e.g. wiping, foaming, spraying, etc. Estimation of reliable exposure is based on (i) data obtained from workplace measurements conducted for this purpose; (ii) historical data of similar products and tasks; and/or (iii) exposure assessment tools in the Biocides Human Health Exposure Methodology (Berger-Preiß et al. 2009; Delmaar and Bremmer 2009; European Chemicals Agency 2017). For the spray application of biocidal products, all three sources of information are available, but corresponding data and models for foam application are lacking. Data on dermal exposure, specifically, are scarce.

Published studies at workplaces deal primarily with the spray application of pesticides and biocides in agriculture and forestry (Garrod et al. 1998; Machera et al. 2003; Marín et al. 2004; Berger-Preiß et al. 2005; Rubino et al. 2012; Mandic-Rajcevic et al. 2015; Roitzsch et al. 2019) or with exposure assessment in industrial settings (Jones et al. 2017; Hebisch et al. 2020). Regarding biocidal products for disinfection or pest control, current scientific literature has featured very little data on inhalation and dermal exposure in the context of spray application (Schipper et al. 1996; Preller and Schipper 1999; Snippe et al. 2001; Rajan-Sithamparanadarajah et al. 2004; Berger-Preiß et al. 2005). Exposure data for foam application are also lacking for these product categories.

As a result of this lack of data, the German Federal Institute for Occupational Safety and Health (BAuA; *Bundesanstalt für Arbeitsschutz und Arbeitsmedizin*), the national authority for the authorisation of biocidal products in Germany, initiated a research project on inhalation and potential dermal exposure to non-volatile active substances in biocidal products during foam application. Measurements were conducted to generate initial data on inhalation and potential dermal exposure by foam application. Comparative data on spray application were collected wherever possible, thereby allowing for the exploration of the potential of foam application as a risk-mitigation measure for the application of non-volatile biocidal products.

## Methods

### Study design

In 2018 and 2019, 26 workplaces or simulated applications of foaming or spraying activities (*n* = 16 foaming, *n* = 10 spraying) were investigated in Germany. These investigations included 12 measurement campaigns in occupational settings, 5 instances of professional pest-control workers at simulated workplaces, 2 applications at simulated workplaces by untrained staff, and 7 simulated applications in model rooms conducted by members of staff not officially trained in spray and foam applications (Fig. S1 in the Supplementary material). Operator-to-scenario assignments are given in Tables 1 and 2; with numbers being allocated to scenarios (#1 to #26) and letters (A to I) to operators. All participants provided written informed consent prior to the study.

**Table 1.** Application parameters and settings for foaming and spraying as well as corresponding inhalation exposure concentrations. For instances in which CsCl was used as an inorganic tracer, the airborne concentration of the active substance (a.s.) is calculated based on its content in the application solution [%] and the airborne CsCl [µg/m³]. The airborne a.s. was determined directly for all other instances. Numerical assignment for scenarios and alphabetical assignment for respective operators.

#	Brief description	a.s. applied (nozzle^a^)	t_app_	Applied BP					Airborne concentration		c_n_	r_app_
				Total amount	Percentage a.s.	Percentage CsCl	Amount of a.s.	Amount of CsCl	a.s.	CsCl	a.s. or CsCl	a.s. or CsCl
			[min]	[kg]	[%]	[%]	[g]	[g]	[µg/m³]	[µg/m³]	[µg/(m³_*_g)]	[g/min]
**Handheld devices (≤3 bar, small-scale applications)**												
Foaming												
1–A	Surface disinfection/ cleaning (workbench); workplace; professional	BAC (screen, 95 mm²)	1	0.325	0.16	0.1	0.52	0.32	0.10	0.16	0.31	0.52
2–A	Surface disinfection/ cleaning (workbench); workplace; professional	BAC (screen, 28.3 mm²)	0.55	0.063	0.16	0.1	0.10	0.06	1.16	1.86	18.4	0.18
9^b^–B	Surface disinfection/ cleaning (tabletops); simulated workplace; untrained staff	BAC (screen, 28.3 mm²)	19	0.559	0.16	0.1	0.89	0.56	0.15	0.25	0.28	0.05
Spraying												
3–A	Surface disinfection/ cleaning (workbench); workplace; professional	BAC (cone, 0.589 mm²)	1	0.096	0.16	0.1	0.15	0.10	5.66	9.06	59.2	0.15
4–A	Surface disinfection/ cleaning (workbench); workplace; professional	BAC (cone, 0.02 mm²)	1.28	0.068	0.16	0.1	0.11	0.07	38.6	61.7	569	0.09
10^b^–B	Surface disinfection/ cleaning (tabletops); simulated workplace; untrained staff	BAC (cone, 0.02 mm²)	17	0.519	0.16	0.1	0.83	0.52	38.3	61.3	73.9	0.05
**Propellant gas-assisted devices**												
Foaming												
11–C	Treatment of wasp nests; simulation in model room; not officially trained staff	pyr (standard and precise application)	3.63	0.549	0.15	-	0.82	NA	NA	1.20	1.46	0.23
12–C	Treatment of wasp nests; simulation in model room; not officially trained staff	pyr (precise application)	3.43	0.372	0.15	-	0.56	NA	NA	0.23	0.41	0.16
27–D	Treatment of wasp nests; simulated work-place; professional	pyr (standard and precise application)	20	0.679	0.105	-	0.71	NA	NA	1.43	2.01	0.04
28–D	Treatment of cock-roach infestation; simulated workplace; professional	pyr (precise application)	20	0.246	2.8	-	6.89	NA	NA	0.60	0.09	0.34
Spraying												
13–C	Treatment of wasp nests; simulation in a model room; not officially trained staff	pyr (standard)	0.38	0.334	0.10	-	0.33	NA	NA	220	659	0.87
**Low- and high-pressure devices (1–6 bar and >10 bar, large-scale applications)**												
Foaming												
5–C	Surface disinfection; simulation in model room; not officially trained staff	BAC (fan, 61.9 mm²)	1.83	10.4	0.16	0.1	16.6	10.4	10.3	16.5	0.99	9.07
6–C	Surface disinfection; simulation in model room; not officially trained staff	BAC (fan, 2.75 mm²; foam cartridge)	3.2	2.9	0.16	0.1	4.64	2.9	9.81	15.7	3.38	1.45
14^c^–E	Surface disinfection (small pigsty), work-place; professional	BAC (fan with screen)	12	225	0.0015	-	5.02	NA	NA	<12.0	<2.39	0.42
15^c^–E	Surface disinfection (henhouse at husbandry); work-place; professional	BAC (fan with screen)	12	230	0.049	-	115	NA	NA	<12.0	<0.10	9.58
18–F	Surface disinfection (sauna); workplace; professional	BAC (foam gun)	5.85	0.15 of concentrate	0.0378	-	11.4	NA	NA	<24.4	<2.14	1.95
20–G	Surface disinfection (sauna); workplace; professional	BAC (foam gun)	5.2	0.15 of concentrate	0.0378	-	11.4	NA	NA	<27.4	<2.40	2.19
21^c^–I	Surface disinfection (pigsty at husbandry); workplace; professional	BAC (fan)	12	2 of concentrate	0.04	-	148	NA	NA	9.33	0.06	12.3
24–D	Surface treatment (walls); simulated workplace; professional	BAC (screen, 75.4 mm²)	20	9.5	1.5	0.1	189	10.2	0.69	9.60	0.07	9.45
25–D	Surface treatment (walls); simulated workplace; professional	BAC (fan, 2.75 mm²; foam cartridge)	31	8	1.5	0.1	159	8.4	27.0	385	3.39	5.13
Spraying												
7–C	Surface disinfection (wall); simulation in model room; not officially trained staff	BAC (fan, 7.8 mm²)	0.92	12	0.16	0.1	19.2	12.0	48.5	77.5	4.04	20.90
8–C	Surface disinfection (wall); simulation in model room; not officially trained staff	BAC (fan, 2.75 mm²)	1	0.8	0.16	0.1	1.28	0.8	26.1	41.8	32.6	1.28
16^c^–E	Surface disinfection (henhouse at husbandry); work-place; professional	BAC (fan)	5	270	0.049	-	135	NA	NA	141	1.0	27.00
17^c^–E	Surface disinfection (small pigsty), workplace; professional	BAC (fan)	5.83	100	0.176	0.087	132	87	256	348	2.64	22.6
19–H	Surface disinfection (public swimming pool); workplace; professional	BAC (fan)	16	38	0.189	-	64.8	-	-	30.0	0.46	4.1
26–D	Surface treatment (walls); simulated workplace; professional	BAC (cone)	12	5	2.0	0.1	99.4	5.4	49.9	998	10.0	8.28

BP - biocidal product (mixture of the active substance and co-formulants) applied during the task, either diluted or as supplied/purchased; a.s.—active substance in biocidal product; t_app_ - duration of spraying or foaming application (note for #9 and #10; sampling included both application and wiping; otherwise, only application was considered); c_n_ - exposure concentration normalised to amount of active substance applied during the task; r_app_ - application rate of active substance; BAC - benzalkonium chlorides; pyr - pyrethroid

^a^nozzle type and orifice area;

^b^sampling comprised spray or foam application as well as subsequent wiping activities;

^c^application with high-pressure devices (>10 bar).

**Table 2.** Potential dermal exposure due to foam and spray applications of biocidal products (data rounded to three figures, *n* = 26). Numerical assignment for scenarios and alphabetical assignment for respective operators.

#	Brief description	a.s. applied	t_meas_	Amount of BP applied	Amount of a.s. applied	Absolute exposure			Normalised exposure		
						Coverall	Gloves	Total	Coverall	Gloves	Total
			[min]	[kg]	[g]	[µg]			[mg/kg]		
**Handheld devices (≤3 bar, small-scale applications)**											
Foaming											
1-1^a^– A	Surface disinfection/ cleaning (work bench); workplace; professional	BAC	6.00	0.325	0.52	205 ^e^	10.3	215	394 ^e^	19.9	414
1-2^b^– A						1,810 ^f^	52,800	54,600	3,490 ^f^	102,000	105,000
1^c^– A						2,020	52,800	54,800	3,880	102,000	105,000
2-1^a^– A	Surface disinfection/ cleaning (work bench); workplace; professional	BAC	3.67	0.063	0.10	33.2 ^e^	39.7	72.9	329 ^e^	394	723
2-2^b^– A						289 ^f^	7,870	8,150	2,860 ^f^	78,000	80,900
2^c^– A						322	7,900	8,230	3,190	78,400	81,600
9^c^– B	Surface disinfection/ cleaning (tabletops); simulated workplace; untrained staff	BAC	19.0	0.559	0.89	57.5	24,400	24,500	64.2	27,300	27,300
Spraying											
3-1^a^– A	Surface disinfection/ cleaning (work bench); workplace; professional	BAC	4.5	0.096	0.15	65.9 ^e^	7.62	73.5	431 ^e^	49.8	480
3-2^b^– A						760 ^f^	12,700	13,500	4,970 ^f^	83,300	88,300
3^c^– A						826	12,800	13,600	5,400	83,400	88,800
4-1^a^– A	Surface disinfection/ cleaning (work bench); workplace; professional	BAC	4.45	0.068	0.11	73.7 ^e^	48.7	122	679 ^e^	449	1,130
4-2^b^– A						253 ^f^	5,500	5,750	2,330 ^f^	50,700	53,000
4 ^c^– A						327	5,500	5,870	3,010	51,100	54,100
10^c^–B	Surface disinfection/ cleaning (tabletops); simulated workplace; untrained staff	BAC	17.0	0.519	0.83	327	12,500	12,800	394	15,100	15,400
**Propellant gas-assisted devices**											
Foaming											
11^a^– C	Artificial wasp nests; simulation in model room	pyr^g^	3.63	0.549	0.82	1.78	213	215	2.16	258	261
12^a^– C	Treatment of wasp nests; simulation in model room; not officially trained staff	pyr^g^	3.43	0.372	0.56	81.4	0.295	81.6	146	0.529	147
27^a^– D	Treatment of wasp nests; simulation in model room; not officially trained staff	pyr^g^	20.0	0.679	0.71	600	82.3	683	842	115	957
28^a^– D	Treatment of wasp nests; simulated workplace; professional	pyr^h^	20.0	0.246	6.89	147	427	574	21.3	62.0	83.3
Spraying											
13^a^– C	Treatment of wasp nests; simulation in model room; not officially trained staff	pyr^g^	0.38	0.334	0.33	356	71.8	428	1,070	215	1,280
**Low- and high-pressure devices (1–6 bar and >10 bar, large-scale applications)**											
Foaming											
5^a^– C	Surface disinfection; simulation in model room; not officially trained staff	BAC	1.83	10.4	16.6	55.6	207	263	3.34	12.5	15.8
6^a^– C	Surface disinfection; simulation in model room; not officially trained staff	BAC	3.20	2.9	4.64	127	49.3	176	27.4	10.6	38.0
14^a; d^– E	Surface disinfection (small pigsty), workplace; professional	BAC	12.0	225	5.02	1,000	1,180	2,180	200	235	435
15^a; d^– E	Surface disinfection (henhouse at husbandry); workplace; professional	BAC	12.0	230	115	1,370	297	1,660	11.9	2.59	14.5
18^a^– F	Surface disinfection (sauna); workplace; professional	BAC	5.85	0.15 of concentrate	11.4	752	1,010	1,770	66.0	88.9	155
20^a^– G	Surface disinfection (sauna); workplace; professional	BAC	5.20	0.15 of concentrate	11.4	325	305	630	28.5	26.7	55.2
21^a; d^– I	Surface disinfection (pigsty at husbandry); workplace; professional	BAC	12.0	2 of concentrate	148	7,190	1,460	8,650	48.6	9.84	58.4
24^a^– D	Surface treatment (walls); simulated workplace; professional	BAC	20.0	9.5	189	15,700	68,700	84,400	83.4	364	448
25^a^ – D	Surface treatment (walls); simulated workplace; professional	BAC	31.0	8	159	2,830	1,890	4,720	17.8	11.8	29.6
Spraying											
7^a^– C	Surface disinfection (wall); simulation in model room; not officially trained staff	BAC	0.92	12	19.2	383	330	713	20.0	17.2	37.1
8^a^– C	Surface disinfection (wall); simulation in model room; not officially trained staff	BAC	1.00	0.8	1.28	34.2	22.3	56.5	26.7	17.4	44.1
16^a; d^– E	Surface disinfection (henhouse at husbandry); workplace; professional	BAC	6.00	270	135	1,810	95.9	1,910	13.4	0.71	14.1
17^a; d^– E	Surface disinfection (small pigsty), workplace; professional	BAC	7.00	100	132	10,800	1,440	12,200	81.5	10.9	92.4
19^a^– H	Surface disinfection (public swimming pool); workplace; professional	BAC	16.0	38	64.8	637	12.4	649	9.83	0.19	10.0
26^a^– D	Surface treatment (walls); simulated workplace; professional	BAC	12.0	5	99.4	14,800	4,750	19,600	149	47.8	197

t_meas_ - measurement time for dermal samplers; BP - biocidal product (mixture of the active substance and co-formulants) applied, during the task either diluted or as supplied/purchased; a.s. - active substance in the (diluted) biocidal product applied during the task; normalised exposure - a.s. on the dosimeter relative to the amount of a.s. applied during the task; BAC - benzalkonium chlorides; pyr - pyrethroid

^a^application

^b^wiping

^c^application + wiping

^d^application with high-pressure devices (>10 bar)

^e^coverall without right forearm

^f^right forearm only

^g^quantification of phenothrin

^h^quantification of permethrin

The application process was the primary focus of these investigations; any peripheral tasks, such as mixing, loading, or post-application work, were excluded wherever possible.

#### Active substances in biocidal products.

Commercially available biocidal products were used which contained quaternary ammonium compounds (QACs), specifically benzalkonium chlorides (BACs), or pyrethroids as non-volatile active substances (a.s.). With regard to pyrethroids, phenothrin and permethrin were considered as a.s., regardless of whether the biocidal product contained additional pyrethroids. The composition of the biocidal products (BPs) and the actual application solutions is given in the contextual information within the Supplementary material.

To monitor inhalation exposure, caesium chloride (CsCl) was used as an inorganic tracer in BAC-based applications wherever possible. The usage of this inorganic tracer made it possible to benefit from the highly sensitive Inductively Coupled Plasma-Mass Spectrometry technique.

#### Application scenarios and application devices.

To cover the large range of foam applications at different workplaces, various application scenarios were included in the design of this study, ranging from small-scale disinfection scenarios to the large-scale disinfection of animal housing, and additionally comprised biocide applications commonly performed by pest-control workers. These scenarios required a wide variety of application devices. Generally, the scenarios can be divided into three categories by the devices used:

- handheld devices (I),
- propellant gas-assisted devices (II), and
- low- and high-pressure devices (III).

Handheld devices for small-scale applications (≤3 bar reservoir pressure; e.g., hand-pump/compression sprayers and foamers) were used to treat horizontal surfaces such as table tops. Propellant gas-assisted devices (pressure cans) were used to treat simulated wasp nests and a simulated cockroach infestation. Low- and high-pressure, partly stationary, devices (low-pressure devices with 1 to 6 bar reservoir pressure; high-pressure devices with >10 bar reservoir pressure) were mainly used for large-scale applications, such as for the treatment of floors and walls, including overhead applications. In all large-scale applications, the nozzle was attached to a pressure-application lance.

The application and exposure duration were generally short, with 19 out of 26 scenarios classified as short-term exposures (<15 min) and the remaining seven as medium-term exposures (15 to 31 min). Both foam and spray applications were conducted for direct comparison wherever the setting and application devices allowed.

More detailed information on each application scenario, e.g. duration, amount of biocidal product applied during the task, etc. is given in Tables 1 and 2. Additional contextual information on each application is given in the Supplementary material (“Sampling campaigns - context”).

### Sampling procedures

Sample-collection procedures were developed and comprehensively validated before the field monitoring part of the study was undertaken (see Supplementary material “Sampling procedures”).

To determine exposure to inhalable particles during foaming or spraying, personal sampling was conducted using personal pumps SKC Deluxe 224-PCMTX8 (3.5 l/min, SKC Ltd., Dorset, UK) with inhalable-particle sampling heads (GSP, sampler for inhalable aerosols, ANALYT-MTC Meßtechnik GmbH, Müllheim, Germany) in combination with suitable filters as sampling media (MCE filter for sampling of CsCl; glass fibre filters for sampling of pyrethroids and PTFE filters for sampling of BAC).

The sampling duration was chosen to represent the task-related exposure during foaming or spraying application (CEN European Standard EN 689 2018). Due to the specific aim of the study, an approach for the calculation of a time-weighted average by considering all exposure scenarios for a working shift was not applied.

Potential dermal exposure was captured via the interception technique, using hooded coveralls made of polyethylene (Tyvek^®^) and cotton gloves (Machera et al. 2003; Rubino et al. 2012; Roitzsch et al. 2019; Hebisch et al. 2020). After sampling, the gloves were left intact and the coveralls were cut into eleven segments each for body site-specific quantification (Fig. S2 in the Supplementary material). Dermal exposure of the face and feet was not considered.

With regard to workplace scenarios #1 - A to #4 - A, #9 - B, and #10 - B, the disinfection process also included wiping treated surfaces. For scenarios #1 - A to #4 - A, gloves were changed between application of the application solution and subsequent wiping, producing two data sets. For #9 - B and #10 - B, the specific application scenario did not allow glove samplers to be changed, resulting in a single data set.

#### Determination of active substances and tracers in air and dermal samples.

The determination of BAC and the pyrethroids, phenothrin and permethrin, was based on available guidelines and methods published in peer-reviewed journals (van Boxtel et al. 2016; VDI/DIN 2016). A method developed in-house was used for the extraction and quantification of the inorganic tracer CsCl in some of the air samples.

The analytical methods were developed and validated prior to the field-monitoring portion of the study (see Supplementary material “Determination of non-volatile active substances and tracers in air and dermal samplers”).

### Calculation and statistics

#### Inhalation exposure.

For inhalation exposure, the a.s. was quantified in 12 out of 26 measurements. Unless stated otherwise, for all cases in which inhalation exposure was determined by quantifying the inorganic tracer CsCl, the data were converted into BAC exposure data based on the nominal BAC concentration of the biocidal product or application solution used (Equation 1).


$$\begin{array}{l} c\left(BAC\right)=c\left(CsCl\right).\;\frac{m\%\left(BAC\right)}{m\%\left(CsCl\right)} \end{array}$$ (1)

c (BAC): airborne BAC concentration [mg/m³]

c (CsCl): airborne CsCl concentration [mg/m³]

*m*% (BAC): concentration of BAC in application solution [%]

*m*% (CsCl): concentration of CsCl in application solution in [%]

#### Dermal exposure.

For potential dermal exposure, the a.s. on the dermal samplers was quantified. The results are given as a total amount of BAC or pyrethroid on the individual coveralls and gloves, respectively. As very different amounts of biocidal products were applied in the individual scenarios, the dermal exposure data are also given as data related to the amount of a.s. applied during the task (mg a.s. on dosimeter/kg a.s. applied [mg/kg]). These measures correspond to the “normalised exposure” given in Table 2. Furthermore, to compare the dermal exposure of individual body parts, the normalised data were standardised to the respective sizes of the dosimeters (µg a.s. on one square centimetre of dosimeter material/kg a.s. applied [µg/(kg*cm²)]).

With regard to workplace scenarios #1 - A to #4 - A, #9 - B, and #10—B, wiping treated surfaces led to extremely contaminated forearm segments (see Table S3 in the Supplemental material). To avoid misleadingly high results, the exposure data of these coveralls were broken down. On one hand, coverall data for “application only” were calculated, excluding coveralls #9 - B and #10 - B and the right forearm segments of coveralls #1- - A to #4 - A (*n* = 24). On the other hand, coverall exposure for “application and wiping” were calculated, summing up the exposure of all segments (scenarios #1 - A to #4 - A, #9 - B, and #10 - B, *n* = 6).

To see a possible difference between foaming and spraying with regard to dermal exposure, aerosol deposition on the coveralls was calculated, discounting exposure by foam flakes or splashes. Assuming that direct contact, foam flakes, and splashes lead to random and rather high exposure levels, dermal “exposure by aerosol deposition” was calculated by considering only the exposure of the five least-exposed coverall segments. To prove the validity of this mathematical approach, the correlation between dermal “exposure by aerosol deposition” and inhalation exposure was tested. Where a close correlation can be determined, the five least-exposed coverall segments should reflect the actual level of aerosol deposition.

First, the surface-related burdens [µg/cm²] of the five least-exposed coverall segments were divided by the amount of a.s. applied [µg/(kg*cm²)] and averaged. To compare aerosol-derived dermal exposure and inhalation exposure, the average surface-related burden was multiplied by the whole area of the coverall (3.02 m^2^). The corresponding inhalation exposure [µg] was calculated from the air concentration of the a.s. [µg/m³] multiplied by the average respiratory minute volume [0.021 m³/min] (European Commission 2017) and by the duration of the respective task [min].

As the Shapiro–Wilk test revealed a non-normal distribution, dermal and inhalation exposure data were log-10 transformed prior to linear regression analysis. The Pearson correlation was calculated using a two-tailed test (IBM SPSS Statistics). A probability of error of less than 1% (*P* < 0.01) was considered statistically significant.

## Results

### Inhalation exposure

Table 1 presents all air-monitoring data for foaming and spraying.

**Fig. 1.**
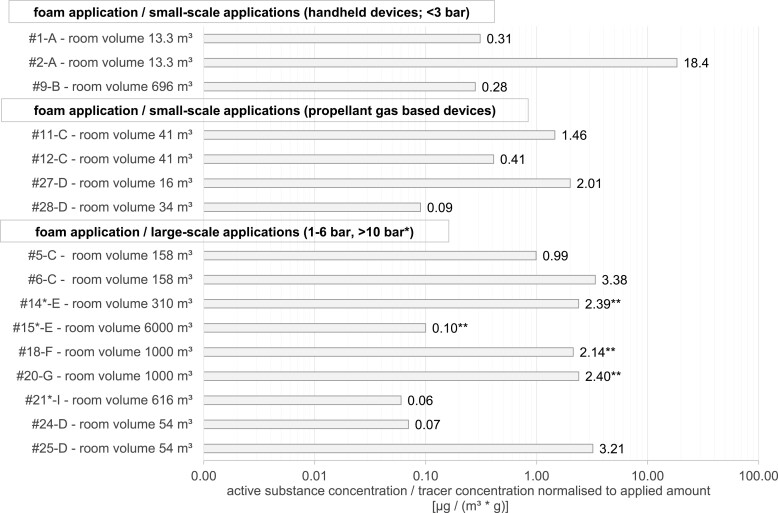
Foam application - inhalation exposure concentration normalised to the amount of a.s./tracer applied during the task. * Applications >10 bar; ** Analytical data were below the limit of quantification, hence values given here were calculated using the limit of quantification.

To allow for a more detailed comparison between individual scenarios as well as for the comparison of foaming and spraying, the exposure levels were normalised to the amount of a.s./tracer applied during the task (Fig. 1 and Fig. S3 in the Supplementary material). Inhalation exposure was associated with the aerosol release rate generated by the application device, specifically the nozzle. Given the low number of exposure scenarios monitored in this study for each nozzle, as well as the short duration of all applications, the calculation of mean values across and within device categories was not considered a viable option for inhalation exposure data.

For foaming across all device categories, the normalised air concentrations varied between 0.06 and 18.4 µg/(m³_*_g). The highest value was found in the category of handheld devices (≤3 bar) for small-scale applications, and the lowest value was ascertained for high-pressure devices (>10 bar). In the category of handheld devices (≤3 bar), with *n* = 3 application scenarios, the factor between the minimum and maximum inhalation exposure levels was 66; this factor was 22 for propellant gas-based devices (*n* = 4 application scenarios). Large-scale applications comprised nine scenarios, of which three were high-pressure applications (>10 bar) and the remaining six were low-pressure applications (1 to 6 bar), whereby the minimum and maximum values differed by a factor of 56.

In the following section, two sub-categories are considered in more detail. Regarding low-pressure foaming devices for large-scale applications, scenarios #24 - D and #25 - D differed by a factor of 46 in their normalised inhalation exposure levels. Even though the application rate was two times lower in #25 - D, the normalised exposure level exceeded that of #24 - D. In both instances, surface treatment with a BAC-based product was conducted using two different devices from the same manufacturer and peripheral parameters (e.g. product, room, and applicant) were identical. A distinct difference was found between the nozzle types used in each application: a screen-based nozzle with an orifice of 75.4 mm² was used in #24 - D, whereas #25 - D used a flat-fan nozzle with an orifice of 2.75 mm² in configuration with a foam cartridge (Fig. S4 in the Supplementary material).

Regarding propellant gas-based foam applications, two artificial wasp nests were treated in a 41 m³ room (scenarios #11 - C and #12 - C). A notable difference regarding inhalation exposure (factor of 5) was observed between the two scenarios, even though similar application rates (0.23 g/min vs. 0.16 g/min) of the same product were applied. As in the previous comparison, different nozzles were used, namely, a standard foam nozzle and a foam nozzle for precise application (Fig. S5 in the Supplementary material). A foam nozzle for precise application, a plastic tube with a small inner diameter, is a mere extension (approx. 14 cm) of the standard foam nozzle which avoids the break-up of the main foam jet and generates a greater distance between source and receptor. In scenario #11 - C, both nozzles were used, and in scenario #12—C, external and internal treatment of the artificial wasp nests was performed using the foam nozzle for precise application. The higher inhalation exposure observed for scenario #11 - C can most likely be attributed to the use of the standard foam nozzle.

Scenario #27—D included the treatment of six artificial wasp nests and the placing of barriers in a bathroom (16 m³) by a pest-control professional using the same propellant gas-based product as in scenarios #11—C and #12—C. Within the category of “foam cans,” the lowest application rate of biocidal product results in the highest inhalation exposure. In reviewing the context of this scenario, the treatment of six nests in such a small compartment might be considered excessive, and the artificially prolonged 20-min stay of the pest-control worker in the room, in conjunction with the use of both nozzle settings, may explain the observed discrepancy between exposure concentration and the application rate of the biocidal product.

In order to directly compare the corresponding foaming scenarios, spray applications (*n* = 10) were conducted wherever the setting and the application device allowed. Normalised inhalation exposure concentration levels varied between 0.46 and 659 µg/(m³_*_g) across all spraying device categories (Fig. S3 in the Supplementary material). Here, the 1,450-fold difference between the minimum and maximum values is even more pronounced than for all monitored foam applications (factor of 307; Fig. 1).

### Dermal exposure

A detailed breakdown of the exposure on the individual coverall segments and cotton gloves is given in the Supplementary material. Table S1 shows the absolute amounts of a.s. on the individual coverall segments [µg]. Table S2 gives the amounts of a.s. on the coverall segments normalised to the amount of a.s. applied during the task [mg/kg]. Supplementary Table S3 shows the amounts of a.s. on the coverall segments normalised to the amount of a.s. applied during the task and to the segment area size [µg/(kg*cm²)]. Supplementary Table S4 provides the corresponding data for the cotton gloves. The total exposure on the individual coveralls and on the gloves is shown in Table 2, categorising the data by device and application type.

With regard to the dermal exposure data, one must bear in mind the different characteristics and absorbencies of the Tyvek^®^ and the cotton material. The higher absorbing capacity of the cotton gloves might have influenced the results, even if the cotton gloves were damp but never wet or soaked after sampling.

The disinfection process at workplace scenarios #1—A to #4—A, #9—B, and #10—B also included wiping treated surfaces. For scenarios #1 - A to #4 - A, gloves were changed and the data were assessed separately, reflecting either application only, wiping only, or both, application and wiping. In contrast, scenarios #9—B and #10—B reflect both, applying the diluted biocidal product and wiping the surface, as the application type did not allow for glove samplers to be changed. Table S5 (in the Supplementary material) shows the calculated arithmetic means, medians, and ranges of the categorised dermal exposure data.

Foam application of biocidal products across all device categories results in absolute exposure levels on the coveralls with a mean value of 2,030 ± 4,220 µg a.s. (median: 325 µg; range: 1.78 to 15,700 µg) (application only, *n* = 15). Relative to the applied amount of a.s., the mean exposure of the coveralls was 148 ± 227 mg/kg (median: 48.6 mg/kg; range: 2.16 to 842 mg/kg). The absolute amounts of the respective a.s. on the gloves differ substantially with a mean value of 5,060 ± 17,600 µg a.s. (median: 297 µg; range: 0.295 to 68,700 µg) (application only, *n* = 15). Relative to the applied amount of a.s., hand exposure was quantified as 107 ± 137 mg/kg (median: 26.7 mg/kg; range: 0.529 to 394 mg/kg). Summed up (coverall and gloves), foam application of biocidal products results in a total dermal exposure of 7,080 ± 21,500 µg a.s. (median: 630 µg; range: 72.9 to 84,400 µg) and 256 ± 287 mg/kg (median: 147 mg/kg; range: 14.5 to 957 mg/kg), respectively, based on the applied amount of a.s. (data are also presented in tabular form in Table S5 of the Supplementary material).

Spray application of biocidal products results—across all device categories—in absolute exposure levels on the coveralls with a mean value of 3,220 ± 5,550 µg a.s. (median: 383 µg; range: 34.2 to 14,800 µg) (application only, *n* = 9). Relative to the applied amount of a.s., the mean exposure of the coveralls is 275 ± 376 mg/kg (median: 81.5 mg/kg; range: 9.83 to 1,070 mg/kg). The mean absolute amounts of the respective a.s. on the gloves are 753 ± 1,570 µg (median: 71.8 µg; range: 7.62 to 4,750 µg) (application only, *n* = 9). Relative to the applied amount of a.s., hand exposure was 89.8 ± 150 mg/kg (median: 17.4 mg/kg; range: 0.192 to 449 mg/kg). Summed up, spray application of biocidal products results in total exposure (coverall and gloves) of 3,970 ± 7,030 µg a.s. (median: 649 µg; range: 56.5 to 19,600 µg) and of 365 ± 500 mg/kg (median: 92.4 mg/kg; range: 10.0 to 1,280 mg/kg), based on the applied amount of a.s. (data are also presented in tabular form in Table S5 of the Supplementary material).

**Fig. 2.**
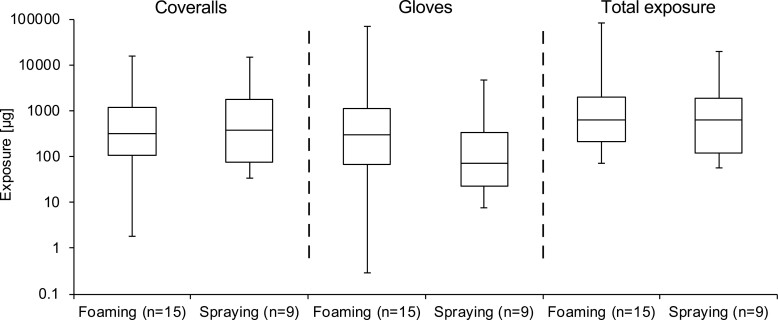
Potential dermal exposure on the coveralls and gloves (application only) for foam and spray applications, respectively. The box-plot visualisation reflects absolute exposure [µg]; the sample size *n* reflects different application scenarios, but not necessarily individual operators.

Comparing the absolute (Fig. 2) and normalised (Fig. S6 in the Supplementary material) dermal exposure data of foam and spray applications, no differences were observed between the two application types (data are given in Table S5 of the Supplementary material).

With regard to the different categories of application devices, regardless of application type, the lowest absolute dermal exposure levels were observed for handheld devices (small-scale applications) and the highest exposure for low- and high-pressure devices (large-scale applications) (Fig. S7 and Table S5 in the Supplementary material). Normalising the exposure data to the amount of a.s. applied during the task shows a contrary finding (Fig. S8 and Table S5 in the Supplementary material).

Even if no reduction in potential dermal exposure could be found when switching from spray to foam application (Fig. 2 and Supplementary Fig. S6), the question remains as to whether the reduction of the aerosol levels observed in the air (see Discussion) could also be interpreted from the dermal data, more precisely from the coverall exposure data. In a mathematical approach, only those coverall segments were considered for which random exposure sources beyond aerosol exposure - as direct contact to the biocidal product/treated surfaces or indirect contact via splashes and foam flakes - could be excluded. The five least-exposed coverall segments were assumed to be ‘contaminated’ solely due to aerosol deposition (see also “Calculation and statistics - dermal exposure”).

**Fig. 3.**
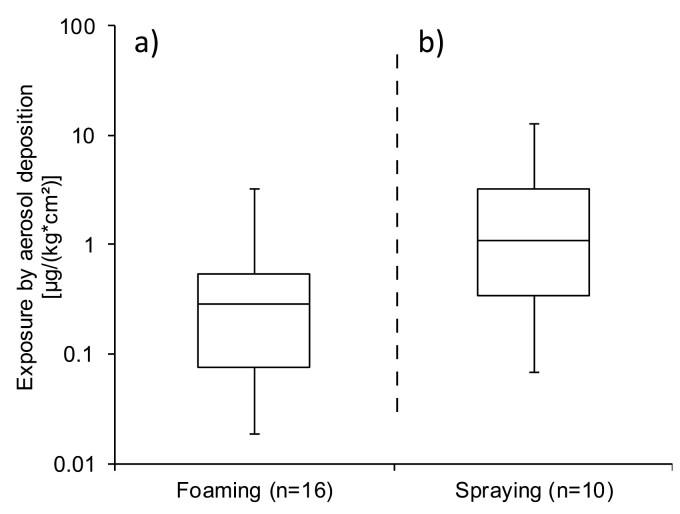
Dermal exposure on the coveralls for foam and spray applications, respectively. The box-plot visualisation reflects the dermal exposure by aerosol deposition, calculated from the means of the five least-exposed coverall segments. The sample size *n* reflects different application scenarios, but not necessarily individual operators.

The data are shown in a log-log graph in Fig. S9 in the Supplementary material. The correlation analysis, which was performed to address the question of whether dermal exposure caused by aerosol deposition correlates with inhalation exposure, revealed a strong and significant relationship. This correlation allows for the comparison of the dermal “aerosol deposition” between foam and spray applications. The data are given in a box-plot diagram (Fig. 3) which shows a trend towards reduced dermal exposure when switching from spraying (b) to foaming (a), assuming aerosol deposition is the sole source for dermal exposure.

**Fig. 4.**
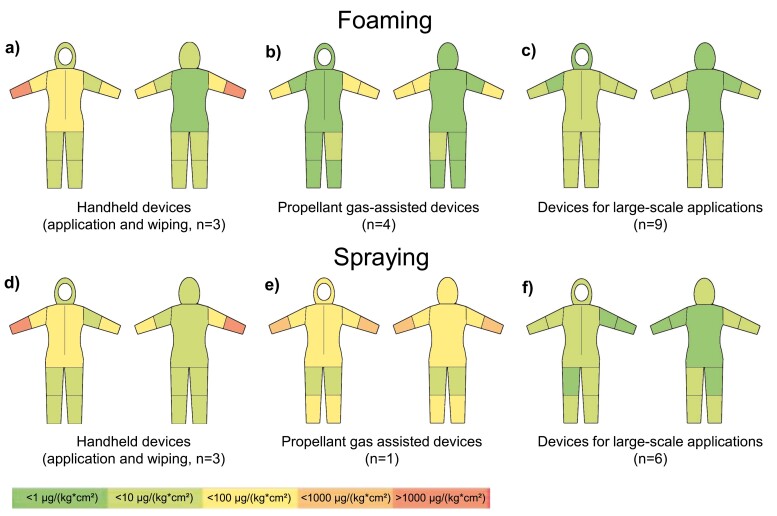
Graphical illustration of exposure patterns on the body. The measured exposure levels to the a.s. per cm² of coverall were additionally normalised to the amount of a.s. applied during the task. The median values of all exposure levels, as related to the foam and spray applications, respectively, were colour-coded. Green shades represent low exposure levels, while orange and red shades indicate higher exposure levels.

Moreover, application-specific exposure patterns on the body were calculated based on the exposure of the different coverall segments. A colour-coded representation of the dermal exposure patterns is given in Fig. 4. The measured exposures of the a.s. per cm² of coverall were normalised to the amount of a.s. applied during the task [µg/(kg*cm²)]. Green shades represent low exposure levels, while orange and red shades indicate higher exposure levels.

Disinfection of worktops and tables using handheld foaming and spraying devices for small-scale applications (both application and wiping) resulted in similar exposure patterns for foaming (a) and spraying (d). The upper front side of the body (breast/stomach) and the forearms were highly exposed, whereas the rest of the body was only slightly affected. While the exposure of the forearms was attributed to wiping disinfected surfaces, the exposure of the upper front part of the body might be explained by aerosol and droplet deposition during and after product application as well as by direct contact, as the operator in scenarios #1 to #4 was relatively short and touched the tables’ edges with the upper front portion of his body. The fact that the a.s. was deposited on the upper front part of the coverall but not on the legs indicates that the treated worktops acted as a barrier for direct exposure.

The treatment of artificial wasp nests and a simulated cockroach infestation with propellant gas-assisted foaming and spraying devices (pressure cans) (*n* = 5) resulted in a heterogeneous distribution of the a.s. on the dermal samplers. With foam application (b; #11 - C, #12 - C, #27 - D, and #28 - D), the exposure of individual body parts was associated mostly with foam flakes landing on the dermal samplers at random, leading to higher exposure levels. The forearm segments, which were located in close proximity to the foam stream emitted from the nozzle, showed slightly elevated mean exposure levels. Spraying of a pyrethroid-containing product (e) was carried out only once (#13—C), which showed higher normalised exposure [mg/kg] compared to the corresponding foam application. The distribution pattern on the coverall showed the highest levels on the forearms.

Foam and spray applications of biocidal products using low- and high-pressure devices for large-scale applications resulted in relatively even mean distribution patterns with no obvious differences between foaming (c) and spraying (f). The front of the body and the legs were slightly more affected, which may have resulted from the application solution splashing back from the treated surfaces (walls/floors).

Moreover, application-specific exposure of the hands should be discussed. With handheld foaming and spraying devices for small-scale applications (application only, #1 - A to #4 - A), hand exposure lies between 19.9 and 449 mg a.s./kg a.s. applied. Considering both application and wiping (#1 - A to #4 - A, #9 - B, and #10 - B), hand exposure was many times higher (15,100 to 102,000 mg a.s./kg a.s. applied). These exposure levels were associated with the manual wiping of treated surfaces with paper tissues. Moreover, the individual data for hand and forearm exposure correspond with the observed dominant hand of the operator (#1 - A to #4 - A: right-handed wiping; #9 - B and #10 - B: mostly left-handed wiping).

With propellant gas-assisted foaming and spraying devices (pressure cans), the normalised hand exposure differed widely and lied between 0.529 and 258 mg a.s./kg a.s. applied. The highest value (#11 - C) resulted from foam-flake deposition on both hands because the operator did not use the extension tube for precise application when treating the outside of the nest, as was used in scenarios #12 - C, #27 - D, and #28 - D. Without this extension tube, his hands were closer to the foam stream emitted from the standard foam nozzle. Furthermore, the similarly high exposure after spraying (#13 - C; 215 mg a.s./kg a.s. applied) resulted from the hands’ close proximity to the standard spray nozzle (fan) used for application.

With foam and spray applications of biocidal products using low- and high-pressure devices for large-scale applications, 0.19 to 364 mg a.s./kg a.s. applied were found on the hands. The range of hand exposure is wide and reflects the different workplace settings within this device category. A variety of large-scale application devices was used and led to equally broad hand-contact possibilities, such as the adjustment of nozzles or the transport of application devices or water hoses. Such demonstrative contacts were only observed in some measurements, such as #14 - E, where the operator moved built-in pigsty partitions that had already been foam-treated during the application process or #25 - D, where the operator refilled the device reservoir.

## Discussion

### Inhalation exposure

To our knowledge, no workplace data have yet been published on inhalation exposure during the foam application of biocidal products. Inhalation exposure to non-volatile a.s. may be driven by the aerosol-release parameters determined by the foam nozzle. Even though the data hereby presented include only an initial and non-representative sample of foaming scenarios and devices, preliminary conclusions may be drawn: the use of devices intended for large-scale foam applications results in higher inhalation exposure levels [µg/m³] than foam applications with handheld devices. This observation does not consider contextual information regarding mass flow, concentration of a.s. in the application solution, applied amount, and other factors. The selected examples indicate that inhalation exposure concentrations during foam applications are influenced by application rate and nozzle geometry, as well as specific workplace settings (see Supplementary material).

Until sufficient contextual information and appropriately large data sets become available, these determinants inhibit a comparison between our results and already published data on inhalation exposure by spraying.

However, after reviewing more than 1,000 documents, Gartiser and Jäger (2013) identified “foam-spray nozzles” as one risk-mitigation measure (RMM) for the application of biocidal products, among others. Even though their focus was the risk of environmental exposure, changing to foam application might also offer a number of advantages over spraying in the context of occupational health and safety. Within a set of initial experiments, Schwarz et al. (2015) showed that changing from spraying to foaming leads to significantly reduced aerosol emissions. The inhalation exposure data obtained during this study confirm this finding, showing that changing from spray to foam application reduces inhalation exposure to the airborne non-volatile substances used in biocidal products.

Exposure data from foam and spray applications obtained within this study, conducted as direct comparisons, were used to quantify the observed exposure reduction by calculating reduction factors (RFs), the ratio between normalised inhalation exposure levels in µg/(m³*g) for corresponding foam and spray applications (Fig. S10 in the Supplementary material).

Across all device categories, the RF varies between 1.2 and 1,607 and shows in all scenarios a reduction in inhalation exposure when changing from spraying to foaming. The wide differences in the RF are due to the variations found in foam and spray applications, as there are innumerable workplace scenarios, application devices, nozzle combinations, and other contextual considerations for both application types. However, in considering the three device categories (handheld devices, propellant gas-assisted devices, and low- and high-pressure devices for large-scale applications), it is notable that the highest exposure reductions were observed with propellant gas-assisted devices, characterised by RFs of 451 and 1,607. Exposure reduction for the other two device categories was markedly lower with RF 31 to 264 for handheld devices and 1.2 to 132 for devices for large-scale applications. The data and findings presented here would benefit from future investigations.

As an outcome of #17 - E (spraying) and #14 - E (foaming), an RF of nearly 1 was determined using a high-pressure device in a small facility, specifically a pigsty (310 m³). This might be considered a somewhat ambitious if not outright unsuitable approach. The disproportionately high mass flow provides conditions for a very quick distribution of aerosol in the comparatively small space, diminishing any intended RMM associated with a change from spraying to foaming.

### Dermal exposure

The collected data on potential dermal exposure underline the findings of other studies (Schipper et al., 1996; Machera et al., 2003; Berger-Preiß et al., 2005) that the main route of occupational exposure during the foam and spray application of biocides is dermal, and that dermal exposure is complex and difficult to predict. Generally, dermal exposure can be caused by direct contact with the biocidal product and treated surfaces or by aerosol deposition on the body. In the workplace settings investigated in this study, direct contact with the biocidal product occurred mainly in the form of foam flakes or product splashes landing on the operators’ bodies. Contact with treated surfaces was also observed, for example, with handheld foaming and spraying devices for small-scale applications. In these cases, hand exposure was clearly attributed to wiping treated surfaces with paper tissues.

To our knowledge, no data were published on workers’ dermal exposure in the context of the foam application of biocidal products. The exposure data obtained within this study are, therefore, compared with spray applications in comparable workplace settings.

For small-scale applications using handheld devices, the average absolute hand exposure in this study (both application and wiping) is higher than the hand exposure levels among cleaners in medical settings as reported by Schipper et al. (1996). They reported an average potential hand exposure of up to 3,000 µg of *o*-phenylphenol and up to 3,500 µg of *o*-benzyl-*p*-chlorophenol (geometric means). The mentioned difference might primarily result from different sampling procedures, as Schipper et al. (1996) used the hand-wash approach to quantify hand exposure. The absorbency of the cotton gloves used in our study might be responsible for the higher exposure data. Similar to our results, Schipper et al. (1996) found inhalation exposure to be negligible compared to dermal hand exposure.

This study found high exposure levels on the hands and forearms as a result of the manual dispersion of disinfectant products by wiping, confirming the findings by Rajan-Sithamparanadarajah et al. (2004), which described higher forearm exposure compared to the rest of the body and even higher levels of hand exposure. These authors also describe the correlation between exposed body parts and applicators’ dominant hands.

Potential dermal exposure by large-scale applications of QAC-containing disinfectants was also studied by Preller and Schipper (1999), measuring inhalation and dermal exposure in slaughterhouses and the meat-processing industry. The biocidal products were applied using low- or high-pressure spraying devices; foam application was not measured. Dermal hand exposure was quantified by means of skin wash to capture actual exposure. This major difference in sampling technique between this study and the work of Preller and Schipper (1999) inhibits a direct comparison between the data sets on hand exposure. The potential exposure of the rest of the body was measured using coveralls and was much larger (88,200 ± 117,000 µg; range: 2,300 to 342,000 µg) when compared to our data for low- and high-pressure application devices. This difference might result from the larger amounts of a.s. applied, but these amounts are not given by Preller and Schipper (1999). The distribution of exposure across the body, as described by Preller and Schipper (1999), is similar to our data, with the lower legs usually exhibiting the highest exposure levels, followed by the upper legs.

In another study, Snippe et al. (2001) quantified respiratory and potential dermal exposure to disinfectants during the spray disinfection of cattle trucks using low-pressure devices. Alkyldimethylbenzyl ammonium chloride was quantified in hand-wash samples (no gloves were worn during disinfection) and on sampling coveralls. Hand exposure was determined to be 292 ± 384 µg a.s. (*n* = 44), and coverall exposure was 1,349 ± 1,409 µg (*n* = 15), with the highest exposure levels found on the lower legs and the hands. The data reported by Snippe et al. (2001) were lower than the dermal exposure data of this study, as less a.s. was applied when disinfecting the cattle trucks.

Berger-Preiß et al. (2005) examined spray applications of pyrethroids in workplace settings using devices for large-scale applications and also published data from food- and feed-area disinfection and from veterinary hygiene. Potential dermal exposure was determined using pads and cotton gloves. The exposure on the body (range: 62.0 to 95,640 µg; *n* = 8) was in the same order of magnitude as our data, while potential dermal hand exposure was considerably higher (range: 6,100 to 53,800 µg; *n* = 2 (data on actual exposure, measured under protective gloves, were omitted)).

Our data show remarkable differences between different device categories, with handheld foaming and spraying devices for small-scale applications leading to the highest exposure levels per kilogram of a.s. applied. Particularly with regard to the relatively small number of operators and experimental replications, it should be noted that, aside from the device categories, inter- and intra-individual differences in behaviour might have had a large influence on individual exposure.

The comparable dermal exposure levels observed for foaming and spraying could be attributed to the fact that exposure from direct contact with the biocidal product or treated surfaces is equally probable with either application technique; moreover, direct contact contributes more significantly to overall dermal exposure than aerosol deposition. If aerosol deposition were solely responsible for dermal exposure, a noticeable difference between foaming and spraying would emerge, even if the difference is - due to the low number of measurements - not statistically significant.

## Conclusion

This study presents initial data on inhalation and potential dermal exposure by foam application of biocidal products in occupational settings as well as comparative data sets for corresponding spray applications. Due to previously missing data, it has become essential to generate valid data of biocidal products. For both, the real-world workplaces and simulated workplace scenarios, comprehensive data for the assessment of inhalation and potential dermal exposure were obtained using validated sampling procedures and analytical methods.

The results of this study demonstrate that changing from spraying to foaming may reduce inhalation exposure to non-volatile a.s. in biocidal products under real occupational conditions. However, exposure reduction varies due to a number of factors, particularly nozzle geometry, product flow rate, and room volume, all of which affect aerosol generation and distribution for both application types.

Moreover, the data of this study revealed that potential dermal exposure accounts for a much higher burden to applicators in occupational settings compared with inhalation exposure. We were able to show that a smaller proportion of dermal exposure is very well correlated with the deposition of the aerosols and that switching from spray to foam application tends to reduce this exposure. Nevertheless, the majority of dermal exposure occurred due to accidental and less-controlled exposure by foam flakes, splashes, and direct contact with the biocidal product/treated surfaces. A reduction in dermal exposure may only be achievable on a case-by-case basis by a combination of structural adjustments and behavioural prevention.

With the data presented herein, an initial, albeit incomplete, database is now available to support the exposure assessment. Looking ahead, further studies on inter- and intra-individual differences in exposure due to behavioural differences would be desirable. Such studies would broaden the database and make an important contribution to exposure assessment as well as to the authorisation of biocidal products applied by foaming.

## Supplementary Material

wxad037_suppl_Supplementary_Material

## Data Availability

The data underlying this article are available in the article and in its online Supplementary material.
