# Supplementary material for: Inhalation and dermal exposure to biocidal products during foam and spray applications
Source: Ann Work Expo Health. 2023 Jul 8;67(7):858–75. doi: 10.1093/annweh/wxad037 (PMC10410488; doi:10.1093/annweh/wxad037)
Supplement: wxad037_suppl_Supplementary_Material [file wxad037_suppl_supplementary_material.pdf]

Supplementary material to the original research paper:

Inhalation and dermal exposure to biocidal products during foam and spray applications

Anja Schäferhenrich[1]*, Katharina Blümlein[2]*, Wolfgang Koch[2], Stefan Hahn[2], Katharina Schwarz[2], Ulrich Poppek[3], Ralph Hebisch[3], Urs Schlüter[3], Monika Krug[3], Thomas Göen[1#]

Abbreviations

BAC	Benzalkonium chloride
a.s.	Active substance
BP	Biocidal product
GC-MS	Gas chromatography-mass spectrometry
GSP	German: *Gesamtstaubprobenahmesystem*; engl: Total dust sampling system
ICP-MS	Inductively coupled plasma-mass spectrometry
ISTD	Internal standard
MCE	Mixed cellulose ester
PBO	Piperonyl butoxide
PTFE	Polytetrafluoroethylene
Pyr	Pyrethroides
QAC	Quaternary ammonium compounds
RF	Reduction factor
RMM	Risk-mitigation measure
TWA	Time-weighted average

Sampling campaigns – context

Workplace sampling – Handheld devices (#1 to #4; #9 and #10)

Product type: Cleaning and disinfection agent
Product: QAC F
Active ingredient: 5–10% Alkyldimethylbenzylammonium chloride (BAC)
2.5–5% Didecyldimethylammonium chloride
Application solution: 2% QAC F (0.16% BAC)
0.1% Caesium chloride (tracer)

Application scenario: #1 – #4
Type: Surface disinfection/cleaning (small-scale)

Operator A: Hygiene specialist (professional user), height: 1.64 m; weight: 78 kg
Personal protective equipment: respiratory protection, visor, cotton sampling gloves over blue nitrile gloves, Tyvek® coverall (sampling media)

Note: The belt, which was equipped with the pumps and sample collectors needed for aerosol measurements, covered small parts of the upper body.

Work procedure for #1 – #4:

In each case, the biocide was applied by one person. The biocide was applied as spray or foam using a hand sprayer or foamer. The product was applied to a small, specific area (3.56 m²; horizontal bench in a laboratory; worktop height: 90 cm), holding the device in the right hand. The subsequent wiping of the surface with a paper tissue was also conducted with the right hand. Gloves were changed between application and wiping.

The operator applied and wiped each surface from right to left. First, the complete application of the disinfectant by foaming/spraying was performed, and the foaming/spraying device was set aside. The entire surface was then wiped with dry tissues. Both sides of the tissue were used. In addition to the work surfaces, the faucet was also foamed and wiped (not included as part of total surface area).

Surfaces for disinfection/cleaning (sketch, not to scale)

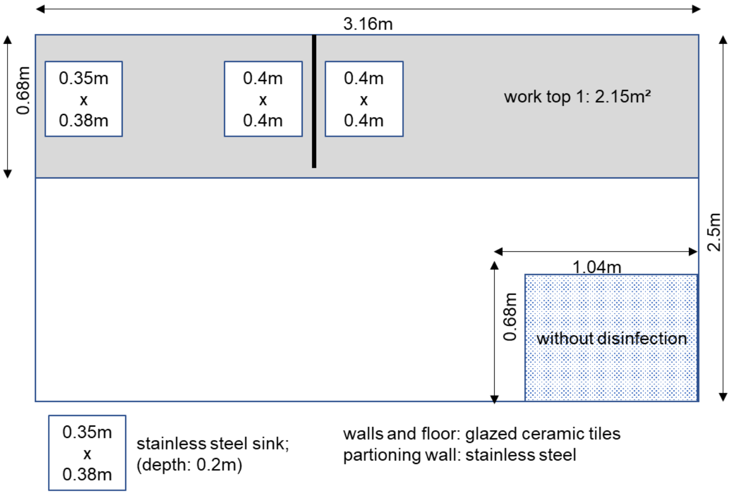

Room volume: 13.3 m³
Air-exchange rate: 8/h
Room temp.: 23–23.5 °C
Rel. humidity: 40–42%

Application scenario: #9– #10

Type: Surface disinfection/cleaning of tabletops (small-scale)

Operator B: Untrained staff; height: 1.84 m; weight: 83 kg

Personal protective equipment: cotton sampling gloves over green nitrile gloves, Tyvek® coverall (sampling media), goggles

Note: The belt, which was equipped with the pumps and sample collectors needed for aerosol measurements, covered small parts of the upper body.

Work procedure: #9 – #10

For the disinfection of several tabletops with a total area of 21 m² (table height: 0.75–0.80 m), the biocide was applied as either spray or foam using a hand-pump sprayer or foamer with a corresponding spray or foam nozzle.

In each case, the biocide was applied by one person. The application solution was applied to each table and wiped with cellulose cloths. Twelve table surfaces were disinfected.

Sketch of the floor plan of the room and disinfected tables (not to scale)

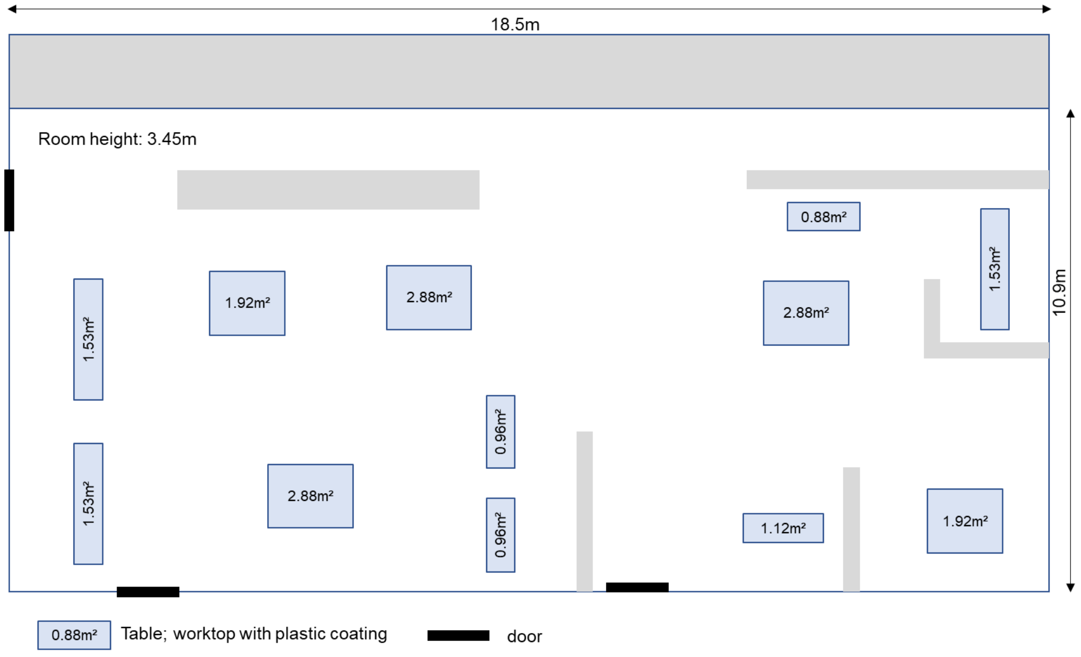

Room volume (net): 696 m³

Air-exchange rate: 2.3/h, including air-supply control via CO_2 sensor

Room temp.: 23.5–25.5 °C

Rel. humidity: 39–40%

#	Application type	Device	Scenario description
#1 - A	Foam	Hand-compression foamer (handheld foaming device with a foam nozzle, up to 3 bar); nozzle: screen, 95 mm^2 orifice	The applied foam was rather liquid and the applied amount was perceived as unusually high by the hygiene specialist; the treated surface was at about the height of the stomach; the operator worked with the right hand (surface disinfection by foam application and wiping); during application, the distance between foam nozzle and hand was about 10 cm and the distance to the treated surface was about 30 cm; contact with biocidal product/treated surfaces: right hand, right forearm, right hip.<br><br>Application duration: 60 s; change of gloves, then wiping with cellulose cloth: 180 s,<br>Total duration of sampling: 360 s
#2 - A	Foam	Hand-pump foamer (handheld foaming device with a foam nozzle); nozzle: screen, 28.3 mm^2 orifice	The applied foam was rather liquid; the treated surface was at about the height of the stomach; the operator worked with the right hand (surface disinfection by foam application and wiping); during application, the distance between foam nozzle and hand was about 5 cm and the distance to the treated surface was about 30 cm; contact with biocidal product/treated surfaces: right hand, right forearm, possibly stomach area.<br><br>Application duration: 33 s; change of gloves, then wiping with a cellulose cloth<br>Total duration of sampling: 220 s
#3 - A	Spray	Hand-compression sprayer (standard)<br>(handheld spraying device, < 3 bar); nozzle: cone, 0.589 mm^2 orifice	Application in a forward-leaning stance; the treated surface was at about the height of the stomach; the operator worked with the right hand (surface disinfection by spray application and wiping); during application, the distance between sprayer head and hand was about 10 cm and the distance to the treated surface was about 20 cm; contact with biocidal product/treated surfaces: right hand, right forearm/elbow, possibly stomach area.<br><br>Application duration: 60 s; change of gloves, then wiping with a cellulose cloth<br>Total duration of sampling: 270 s
#4 - A	Spray	Hand-pump sprayer (handheld spraying device); nozzle: cone, 0.02 mm^2 orifice	Application in a forward-leaning stance; the treated surface was at about the height of the stomach; the operator worked with the right hand (surface disinfection by spray application and wiping); during application, the distance between sprayer head and hand was about 5 cm and the distance to the treated surface was about 15–20 cm; contact with biocidal product/treated surfaces: right hand, right forearm/elbow, possibly stomach area. The operator performed both spray application and wiping from right to left.<br><br>Application duration: 77 s; change of gloves, then wiping with a cellulose cloth<br>Total duration of sampling: 267 s

#	Application type	Device	Scenario description
#9 - B	Foam	Hand-pump foamer (Handheld foaming device); nozzle: cone, 0.589 mm² orifice	Left-handed employee. During application, the distance between nozzle and hand was about 5 cm and the distance to the treated surface was about 30 cm. The employee applied foam to each table and wiped with cellulose cloths, whereby the employee pumped 20x to 80x depending on the size of the table (rather high usage). The applied foam was quite liquid; at one point, the employee rested the right hand on the surface of the table.<br><br>The handheld foaming device was mostly held and operated with the left hand; surface disinfection by wiping was performed with both the right and left hands (each hand was used to wipe about half of each table); the employee pumped 20x to 80x times per table (rather high usage); the applied foam was quite liquid.<br><br>Contact with the biocidal product may have occurred via aerosol, with the treated surface, with wet tissues with the right or left hand during wiping, or when resting the right hand on a table surface (1x) as well as when holding the foaming device with the free hand during application. During application, the forearm of the spraying arm was occasionally supported with the hand of the other arm.<br><br>Application duration: 19 min
#10 – B	Spray	Hand-pump sprayer (handheld spraying device); nozzle: cone, 0.02 mm² orifice	Left-handed employee. During application, the distance between nozzle and hand was about 5 cm and the distance to the treated surface was about 30 cm. Application and wiping was performed with cellulose cloths for each table. Both the left and right hands were used for spraying; in each case, the other hand was used to support the spray bottle; surface disinfection by wiping was performed with both the left and right hands (seven tables with the left hand, five tables with the right), whereby a substantial amount of product was applied; the spray mist was clearly visible.<br><br>Contact with the biocidal product may have occurred via spray mist, with the treated surface, or with wet tissues with the right or left hand during wiping as well as when holding the spray bottle with the free hand during application. During application, the forearm of the spraying arm was occasionally supported with the hand of the other arm.<br><br>No contact was observed between the treated surfaces and forearms or the stomach/pelvic area, as the tables were very low and since chairs/benches stood between the applier and the treated surfaces.<br><br>Application duration: 17 min

Workplace sampling – Propellant gas-assisted devices (#11–#13; #27 and #28)

Product type: Household insecticides
Device: Pressure can

Application scenario	Product	Active ingredient
#11 - C;<br>#12 - C	Insect foam B2	1.5 g/kg Tetramethrin<br>1.5 g/kg 1R-*trans*-Phenothrin
#27 - D	Insect foam B1	3.1 g/kg Tetramethrin<br>1.05 g/kg d-Phenothrin
#13 - C	Insect spray B	4.05 g/kg Tetramethrin<br>1 g/kg d-Phenothrin<br>17.4 g/kg PBO
#28 - D	Insect foam F	28 g/L Permethrin<br>1 g/L Pyrethrine<br>2 g/L PBO

Application scenario #11–#13; #27 and #28
Type: Application of household insecticides (simulations)

Operator C #11–#13: Application specialist (Employee of Fraunhofer ITEM, project-related experience in product application using various devices/techniques); height: 1.85 m; weight: 98 kg

Operator D #27 and #28: Pest-control professional (pest-control professional); height: 1.67 m; weight: 66 kg

Personal protective equipment for operator #11–#13:
Respiratory protection (full-face mask), cotton sampling gloves over blue nitrile gloves, Tyvek® coverall (sampling media)

Personal protective equipment for operator #27 and #28:
Respiratory protection (FFP2 mask), cotton sampling gloves over blue nitrile gloves, Tyvek® coverall (sampling media), work shoes

Note: The belt, which was equipped with the pumps and sample collectors needed for aerosol measurements, covered small parts of the upper body.

Work procedure for #11 and #12
In each case, the biocide was applied by one person. For the simulated treatment of wasp nests using a wasp-control foam, in each case two simulated wasp nests (inflated paper bags) were fixed at a height of 2.20 m in the corner of a model room. A foam can with a standard foam nozzle (fan-shaped product stream with a wide cone of distribution) and a nozzle for precise application were used. For each scenario, two cans of wasp-control foam were used. Foam was first applied to the outside of the nests, which were then filled with wasp-control foam using an application tube.

Work procedure for #13
The biocide was applied by one person. For the simulated treatment of wasp nests using a wasp-control spray, a simulated wasp nest (inflated paper bag) was fixed at a height of 2.20 m in the corner of a model room. A can with a standard spray nozzle (fan) was used. One can of wasp-control spray was applied. The outside of the nest was sprayed.

Surfaces for treatment (sketch, not to scale)

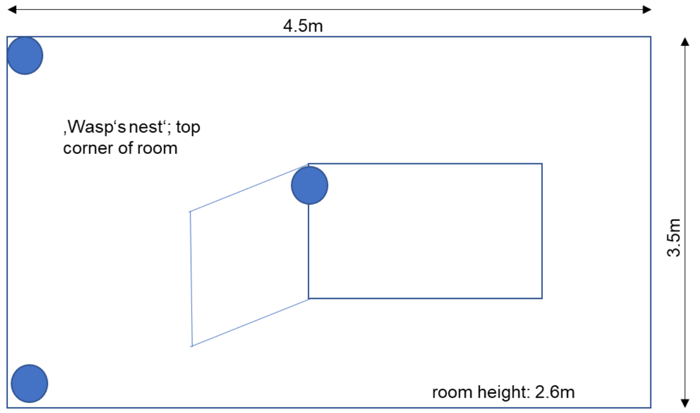

Room volume (net): 41.0 m^3
Air-exchange rate: No active air exchange
Room temp.: 18.5–22.0 °C
Rel. humidity: 40–48.5%

Work procedure for #27
The biocide was applied by one person. For the simulated treatment of six wasp nests using a wasp-control foam, six simulated wasp nests (inflated paper bags) were fixed at a height of 2.0–2.2 m on the walls of a bathroom in a detached house (no furniture, but standard bathroom appliances). A foam can with a standard foam nozzle and a nozzle for precise application were used. For each nest, two cans of wasp-control foam were applied. Foam was first applied to the outside of the nests, which were then filled with wasp-control foam using an application tube. In addition, a foam barrier was applied at the opening through which the "wasps" could enter the interior of the apartment (in this case: window frames). According to the pest-control professional, one can is usually sufficient to treat 20 nests; in most cases, the nests are not treated externally.

Room volume (net): 16 m^3
Air-exchange rate: No active air exchange
Room temp.: 16 °C
Rel. humidity: 63%

Work procedure for #28
The biocide was applied by one person. For the simulated treatment of a cockroach infestation in an unfurnished room in a single-family home, an active foam barrier was applied. A foam can with a standard foam nozzle and a nozzle for precise application were used. One can of foam was applied. The biocidal foam was applied along skirting boards, ceiling strips, door and window frames, as well as other areas. About 57 m of foam barriers were installed using the nozzle for precise application.

Room volume (net): 34 m^3
Air-exchange rate: No active air exchange
Room temp.: 16 °C
Rel. humidity: 54%

#	Application type	Device	Scenario description
#11 – C	Foam	Pressure can	During external foaming, the employee stood a distance of about 1–2 m from the nest; when filling the nest, the employee stood at a distance of about 0.5 m. The distance between nozzle and hand was about 10–15 cm. The casting distance indicated on the foam can (2–3 m) was not achieved, such that foam landed both on the wall and on the floor. Foam flakes settled on both the right and left gloves.<br><br>Application duration: 218 s
#12 – C	Foam	Pressure can	During external foaming, the employee stood at a distance of about 1–2 m from the nest; when filling the nest, the employee stood at a distance of about 0.5 m. The distance between nozzle and hand was about 15 cm. The first can was held in the left hand and the second can was held in the right.<br><br>Application duration: 206 s
#13 – C	Spray	Pressure can	During spraying, the employee stood a distance of about 2–2.5 m from the nest; the distance between nozzle and hand was about 10 cm.<br><br>Application duration: 23 s
#27 – D	Foam	Pressure can, foam number (ratio of foam volume to liquid volume): 28	The employee worked in a closed room; direct observation of application was not possible due to a corrugated window pane. The following information was inquired after application: the employee worked at a distance of about 1 m from a given nest, whereby it was necessary during application to close the distance with the hand holding the foam can by about 5–10 cm. The distance between foam nozzle and hand was about 10–15 cm. The applied foam was very stable; small foam flakes formed when foaming the simulated wasp nests. A large foam flake landed on the left upper arm and a smaller one landed on the left forearm. Application was directed upwards, sometimes overhead.<br><br>Application duration: 20 min
#28 – D	Foam	Pressure can, foam number (ratio of foam volume to liquid volume): 41	The employee shook the spray can prior to application and placed the tube in direct contact with the wall in order to generate a clean foam line. The distance between foam nozzle and hand was about 10–15 cm. In order to apply a foam barrier to the skirting board, the employee worked in a squatted position/on the knees and held the active arm close to the body. When treating the ceiling strips, the active arm was outstretched and the device held overhead. In addition to skirting boards and ceiling strips, foam barriers were applied around the window and door as well as around connector and light-switch covers, the ceiling-light plinth, and in the crevices around the coat rack.<br><br>The employee mostly held the foam can in the right hand and operated the foam nozzle with the left hand. In order to reach the ceiling strips, the employee used a stool which was moved as the work progressed. At times, the employee used the left hand to push the hood back a bit.<br><br>Application duration: 20 min

Workplace sampling – Large-scale applications with low- and high-pressure devices (#5–#8; #14–#21; #24–#28)

Several different scenarios were considered for large-scale spray and foam application. In the following section, application scenarios are grouped to enable, where applicable, a direct comparison between foaming and corresponding spraying scenario(s).

Application scenario: #5–#8

Product type: Disinfectants

Biocidal product (BP): QAC F

Active substance (a.s.): 5–10% Alkylbenzyldimethylammonium chloride (BAC)

2.5–5% Didecyldimethylammonium chloride

Application solution: 2% QAC F (0.16% BAC)

0.1% CsCl (tracer, spiked)

Type: Disinfection of surfaces (large-scale)

Operator C: Member of staff not officially trained in spray and foam application; height: 1.85 m; weight: 98 kg

Personal protective equipment for the operator: Cotton sampling gloves over blue nitrile gloves, Tyvek® coverall (sampling media), respiratory protection, safety glasses, boots (Dunlop Acifort EN ISO 20345:2011 S5 SRA)

Note: The belt, which was equipped with the pumps and sample collectors needed for aerosol measurements, covered small parts of the upper body.

Work procedure for #5 and #6

In each case, the biocide was applied by one person (foaming). A wall with an area of 22.5 m^2 was disinfected. The QAC-containing foam was applied with a foam nozzle, whereby the operator applied the foam at a distance of about 1.20 m. Wide, horizontal foam layers were applied starting from the bottom-right corner. During application, the foam slid downwards along the vertical wall.

Work procedure for #7 and #8

In each case, the biocide was applied by one person. Spray application was conducted in both upwards and downwards motions, starting at the bottom-right corner of the wall and finishing at the left end of the wall.

Treated area (sketch, not to scale):

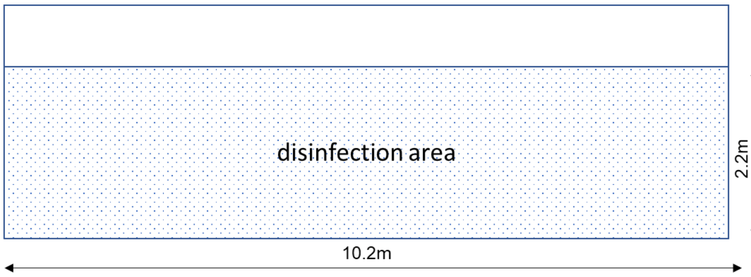

treated area: 22.5m²
Walls and floor: with 2K paint coating (DD)

Room volume:	158 m³
Air-exchange rate:	8/h
Room temp.:	21.0–21.5 °C
Rel. humidity:	45.5–53.5%

#	Application type	Device	Scenario description
#5 – C	Foam	Pressure foamer G; foam nozzle 50/200 (flat-fan nozzle; 61.9 mm² orifice)<br><br>Pressure I: 6.0 bar (foam);<br>foaming air supply: 113 L/min<br>Pressure II: 5.5 bar (pump)	A wall was treated by applying five horizontal foam layers, each at a width of approx. 50 cm. The operator started at the bottom-right corner and finished at the top-left corner. The BAC-containing foam was applied using a foam nozzle. The foaming lance was operated with the left hand. The distance between operator and wall was approx. 1.2 m. The distance between foaming-lance orifice and operator's left hand was approx. 30 cm.<br><br>Exposure/application duration: 110 s
#6 – C	Foam	Pressure foamer P (1–3 bar), flat-fan nozzle (2.75 mm² orifice) with foam cartridge (highly foaming)<br><br>Pressure I: 3 bar (device)<br>Pressure II: 2–2.5bar (application pressure)	A wall was treated by applying six horizontal foam layers. At the beginning (up to 1.5 min), the foam was rather liquid (pressure II: 2 bar). With the fourth foam layer, the foam became thicker. The foaming lance was operated with the left hand, then swapped to the right when applying the fifth foam layer. The right hand was used to carry the pressure foamer (at the beginning and throughout application). The distance between operator and wall was approx. 1.2 m. The distance between foaming-lance orifice and operator's hand was approx. 55 cm.<br><br>Exposure/application duration: 192 s
#7 – C	Spray	Pressure foamer G; spray nozzle 30/40 (flat-fan nozzle; 7.8 mm² orifice)<br><br>Pressure: 5.5 bar (pump)	The wall was treated by spraying in up-and-down motions from the bottom-right corner to the left. The spraying lance was operated with the right hand. The distance between nozzle orifice and operator's right hand was approx. 10 cm.<br><br>Exposure/application duration: 55 s
#8 – C	Spray	Pressure sprayer P (1–3 bar); flat-fan nozzle (2.75 mm² orifice)<br><br>Pressure I: 3 bar (device)<br>Pressure II: 1.5 bar (application pressure)	The wall was treated by spraying in up-and-down motions from the bottom-right to the left. The spraying lance was operated with the left hand. The distance between nozzle orifice and operator's left hand was approx. 55 cm.<br><br>Exposure/application duration: 60 s

Application scenario: #14 – E and #17 – E

Product type: Disinfectants

Biocidal product (BP): QAC E

Active substance (a.s.): 5–10% Alkylbenzyldimethylammonium chloride (BAC)

2.5–5% Didecyldimethylammonium chloride

Application solution #14: 1.32 g/L BAC;

0.1 % CsCl (tracer, spiked);

10% foaming agent/stabiliser

Application solution #17: 2% QAC E (0.16% BAC);

0.1 % CsCl (tracer, spiked)

Type: Disinfection of surfaces (large-scale)

Operator E: Professional (right-handed) with 22 years of experience in large-scale disinfection; height: 1.82 m; weight: 81 kg

Personal protective equipment for the operator: Cotton sampling gloves over blue nitrile gloves, Tyvek® coverall (sampling media), full-face respiratory protection, Wellington boots

Note: The belt, which was equipped with the pumps and sample collectors needed for aerosol measurements, covered small parts of the upper body.

Work procedure for #14 and #17

In each case, the biocide was applied by one person. For the disinfection of a small pigsty (floor area of 124 m²) by foam (#14) or spray (#17) application, the floor and walls (including windows) were treated using a QAC-containing disinfectant. The ceiling and grids between partitions were partially treated.

The pigsty was disinfected starting from the back outer corner of Partition 3, with the operator working his way from back to front, towards the door. The treated surfaces were mainly at the height of the legs (floor and lower wall area), but some surfaces were also at the height of the stomach (fixtures and walls) as well as over the head (upper wall area and parts of the ceiling).

Application was not a continuous process; it was briefly interrupted multiple times when the operator moved between partitions.

Treated area (sketch, not to scale):

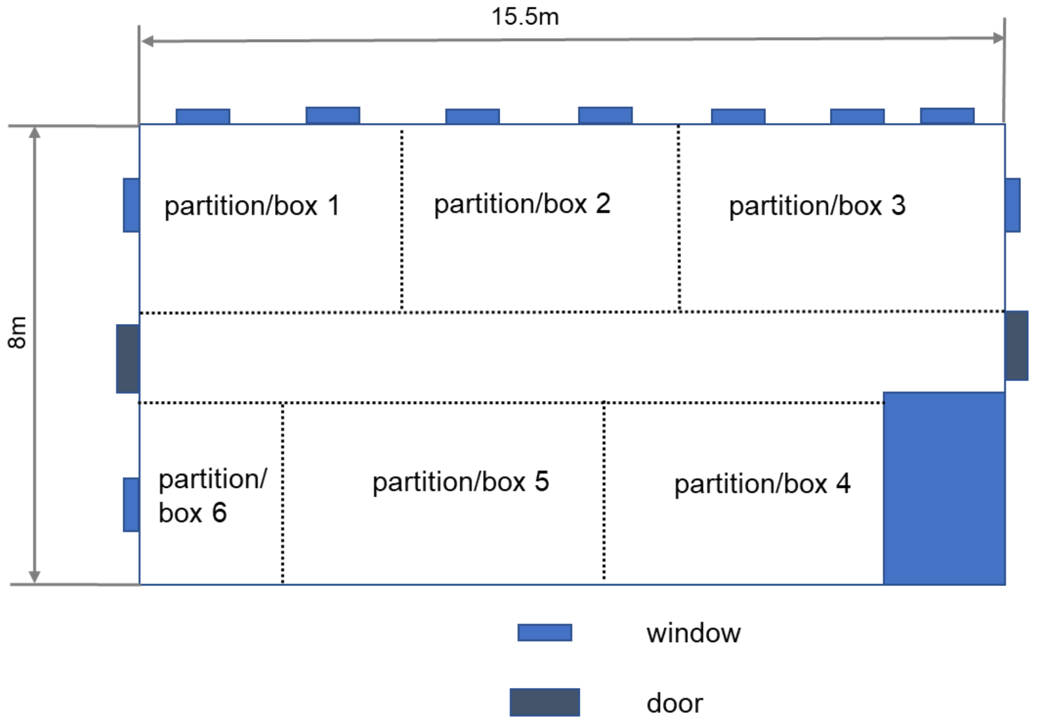

Room volume:	Approx. 310 m³ (height: approx. 2.5 m)
Air exchange rate:	No active air exchange; both doors were open
Room temp.:	Data logger was defective (outdoors: 27–33 °C; indoor estimate: 20 °C
Rel. humidity:	Data logger was defective (outdoors: approx. 75–78%)

#	Application type	Device	Scenario description
#14 – E	Foam	Self-built construction. Water was supplied via pump. Biocidal product, CsCl, and foaming agent were kept in a two-litre reservoir, which was attached to a pressure foam gun (Venturi nozzle).<br><br>Pressure (reservoir pump): 95–100 bar<br><br>Flat-jet nozzle with screen (nominal power: 25 L/min; nominal pressure: 200 bar)<br><br>Ratio of foam-to-liquid volume: 3.5	Two applications were conducted consecutively (1x 7 min; 1x 5 min), whereby the application solution needed to be replenished by study staff between applications. Application was mostly performed in up-and-down motions. The applied foam was rather fluid.<br>The foam lance was primarily held with the right hand, whereby the hand was located at the height of the stomach and breast area. The water hose was pulled/held with the left hand. Occasionally, the left hand was used for spraying or the foam lance was held with both hands. The distance between hand and nozzle was approx. 60 cm. The distance to treated surface was approx. 1–2 m. Study staff helped the operator move equipment. The reservoir was refilled outdoors.<br><br>Treated area: approx. 215 m²<br><br>Contact with biocidal product/treated surface: hands when moving (opening/closing) box grids; the operator briefly stood in the foam spread on the floor; before starting measurement, the operator was already wearing the whole-body dosimeters when he carried a power cable outside to his car; the operator's hands touched the car and water hose in the context of refilling the reservoir with application solution.<br><br>Application duration: 12 min
#17 – E	Spray	Self-built construction. Application solution was supplied from a reservoir via external pump.<br><br>Pressure (reservoir pump): 200 bar<br><br>Flat-fan nozzle (15/10)	Application was mostly performed in up-and-down motions (with occasional horizontal movements) at a rate of approx. 20 L/min.<br>The spray lance was held with both hands (with the left hand on the operating lever); the lance was at the height of the stomach and breast area. The distance between hand and nozzle was approx. 100 cm. The distance to the treated surface was approx. 2 m.<br>Application was not a continuous process; it was briefly interrupted multiple times when the operator moved between partitions. There was not enough prepared application solution to treat the entire pigsty.<br><br>Treated area: approx. 215 m²<br><br>Contact with biocidal product/treated surface: hands when moving (opening/closing) box grids. During application, the operator stood in the sprayed mist; at the start of measurement, the operator was already wearing the whole-body dosimeters when he activated the pump/mixing unit and put on his full-face respiratory protection.<br><br>Application duration: 7 min

Application scenario: #15 and #16

Product type: Disinfectants

Biocidal product (BP): BAC-containing product

Active substance (a.s.): 25 g/L Benzyl-(C12–C16)-alkyldimethylammonium chloride (BAC);

125 g/L Formaldehyde;

250 g/L Glutaral

Application solution #15: 0.5 g/L BAC

Application solution #16: 0.5 g/L BAC

Type: Disinfection of surfaces (large-scale)

Operator E: Professional (right-handed) conducting QAC-based disinfections on a daily basis; height: 1.78 m; weight: 85 kg

Personal protective equipment for the operator: Cotton sampling gloves over blue nitrile gloves, Tyvek® coverall (sampling media), full-face respiratory protection, Wellington boots

Note: The belt, which was equipped with the pumps and sample collectors needed for aerosol measurements, covered small parts of the upper body.

Work procedure for #15 and #16

In each case, the biocide was applied by one person. A henhouse (type: Louisiana) was disinfected using QAC-containing disinfectant. The floor, walls (up to the height of windows at approx. 3 m), doors, and overhead fixtures (automatic water dispenser and feeding lanes, six lanes in total) were treated. The treated surfaces were located at the height of the legs (floor and lower wall areas), at the height of the breast, and overhead (upper wall area, installations (#15 and #16), and stall ceilings (#16)).

The disinfectant was applied either with a pressure foam gun (#15) or a spray nozzle (#16). During application, one of the two doors remained open, resulting in a slight draft.

The operator’s movements are given in the sketch below. During foam application (#15), the operator walked backwards; for movement in Directions 3 and 7, the disinfectant was applied against the draft. During spray application (#16; mainly overhead), the operator walked backwards; for movement in Direction 2, the disinfectant was applied against the draft.

Treated area (sketch, not to scale):

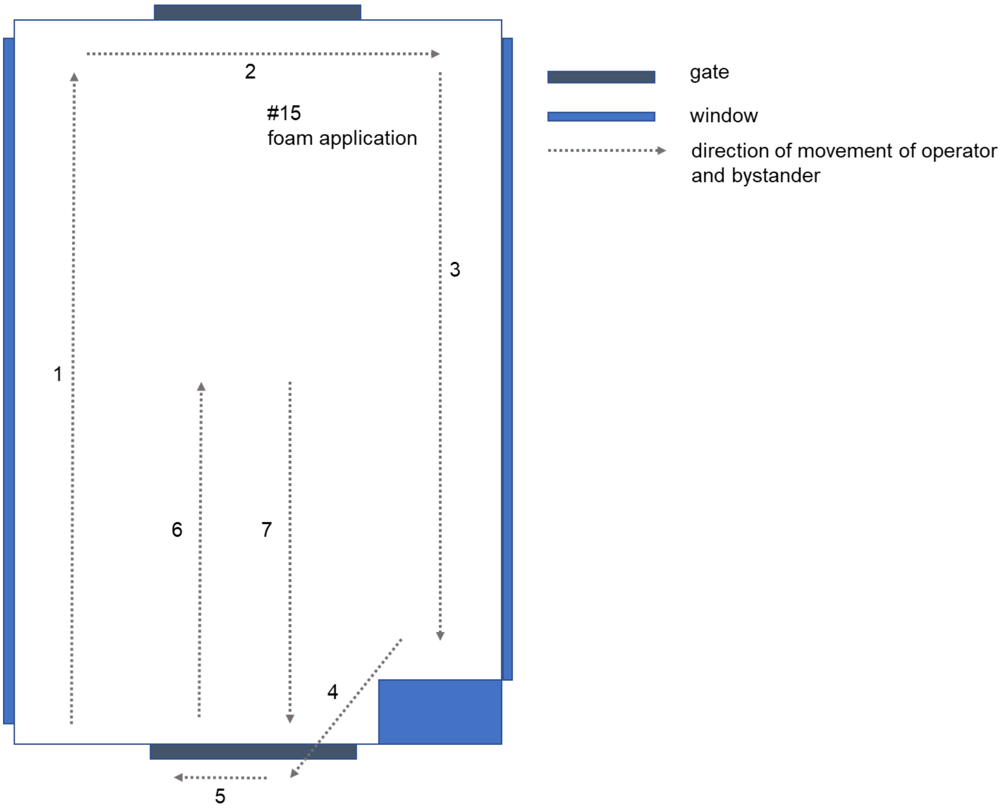

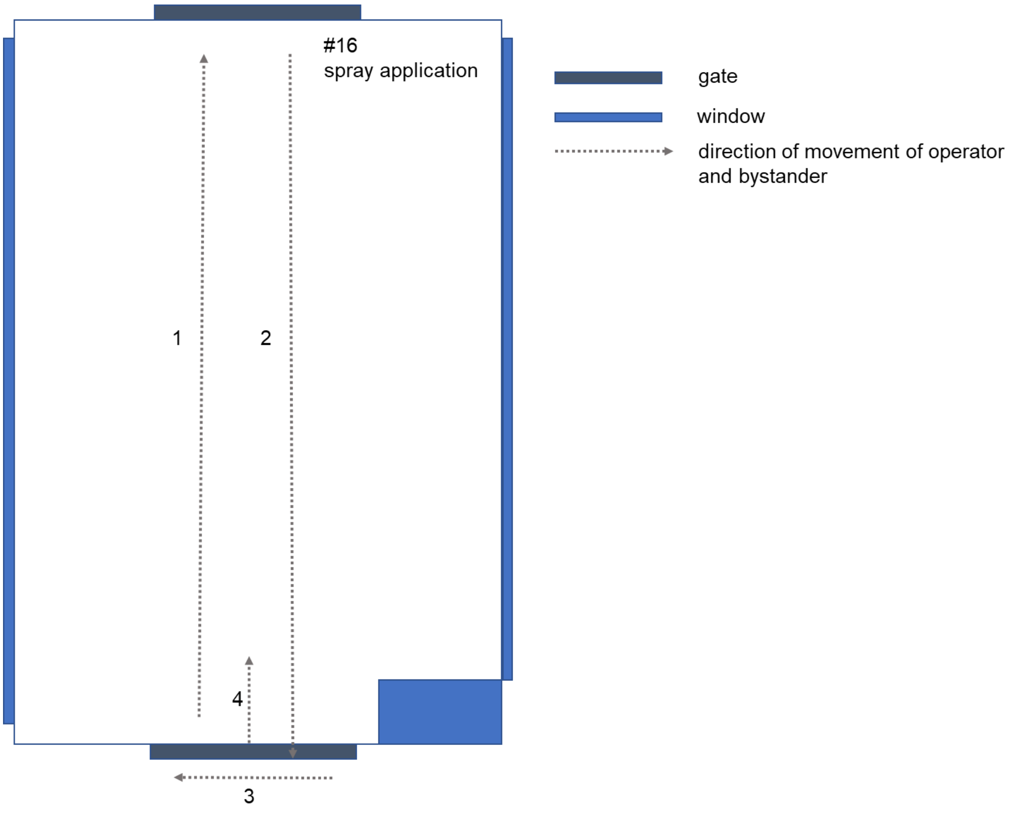

Treated area (sketch, not to scale):

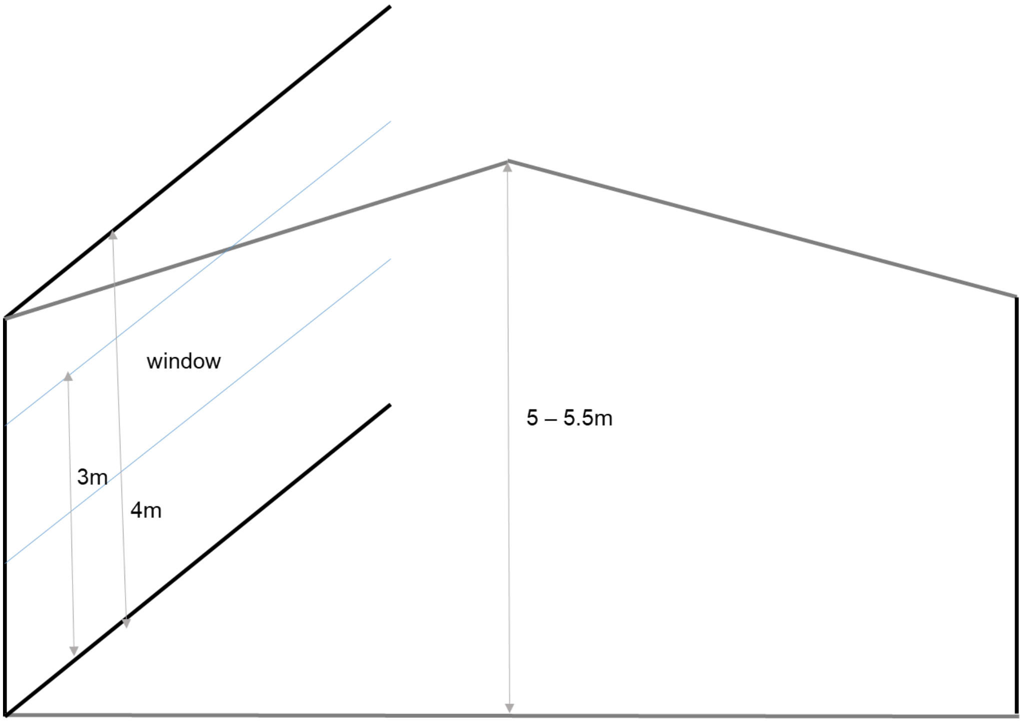

Room volume:	Approx. 6000 m³
Air-exchange rate:	No active air exchange; one door was open
Room temp.:	Data logger was defective (outdoors: 27–33 °C; indoor estimate: 30 °C
Rel. humidity:	Data logger was defective (outdoors: approx. 75–78%)

#	Application type	Device	Scenario description
#15 - E	Foam	Self-built construction. Application solution was supplied via pump. Foaming agent was supplied from a separate reservoir attached to the foaming lance according to the Venturi principle.<br><br>Pressure (reservoir pump): 70–80 bar<br><br>Flat-jet nozzle with screen (nominal power: 25 L/min; nominal pressure: 200 bar)<br><br>Ratio foam-to-liquid volume: 7	Application was performed in both vertical and horizontal motions (occasionally overhead) at a rate of approx. 25 L/min.<br>The applied foam was rather liquid. The casting distance was approx. 4 m, which was not sufficient to treat the ceiling. The foam lance was mainly operated with the right hand and the water hose was pulled/held with the left hand. The distance between hand and nozzle was approx. 0.5 m. The distance to the treated surface was approx. 2–3 m. The walls were disinfected primarily in up-and-down motions; in contrast, the floor and installations were disinfected in horizontal motions from right to left.<br><br>Floor area of henhouse: approx. 1100m²<br><br>Contact with biocidal product/treated surface: The hands came in contact with the foaming lance and water hose. In an initial approach, a pump pressure of 120 bar was applied, causing the hose to burst. The operator was therefore exposed to an aerosol cloud for a short time. After this incident, the cotton gloves (sampling media) were swapped for a clean pair.<br><br>Application duration: 12 min
#16 - E	Spray	Self-built construction. Application solution was supplied via pump.<br><br>Pressure (reservoir pump): 120 bar<br><br>Flat-fan nozzle, type 30 (nozzle capacity: 120 L/min)	Application was performed in both vertical and horizontal motions at a rate of approx. 50 L/min.<br>The casting distance was approx. 8–10 m The spraying lance was mainly operated with the right hand and kept at head level. The sprayed mist was distributed throughout the entire henhouse. The distance between hand and nozzle was approx. 1.4 m. The distance to the treated surface was approx. 3–4 m.<br><br>Floor area of henhouse: approx. 1100m²<br><br>Contact with biocidal product/treated surface: The hands came in contact with the spraying lance and water hose.<br><br>Application duration: 6 min

Application scenario: #18–#20

Product type: Disinfectants

Biocidal product (BP): BAC-containing product

Active substance (a.s.): 7.6% (w/w) Benzalkonium chloride

Application solution #18: 0.4 g/L BAC

Application solution #19: 1.9 g/L BAC

Application solution #20: 0.6 g/L BAC

Type: Disinfection of tiled surfaces (large-scale)

Operator: #18 - F: Professional (right-handed) conducting QAC-based disinfections on a daily basis; height: 1.53 m; weight: 50 kg

#19 - H: Professional (right-handed) conducting QAC-based disinfections once or twice a month, depending on the shift schedule. height: 1.90 m; weight: 105 kg

#20 - G: Professional (right-handed) conducting QAC-based disinfections on a daily basis; height: 1.79 m; weight: 86 kg

Personal protective equipment for operator #18: Cotton sampling gloves over green nitrile gloves, Tyvek® coverall (sampling media), safety glasses, Wellington boots

Personal protective equipment for operator #19: Cotton sampling gloves over blue nitrile gloves, Tyvek® coverall (sampling media), safety glasses

Personal protective equipment for operator #20: Cotton sampling gloves over green nitrile gloves, Tyvek® coverall (sampling media), safety glasses, Wellington boots

Note: The belt, which was equipped with the pumps and sample collectors needed for aerosol measurements, covered small parts of the upper body.

Work procedure for #18–#20

All three scenarios took place in different locations of a public indoor swimming pool (#18 and #20 in the sauna; #19 in the tiled area next to the pool). In each case, the biocide was applied by one person.

#18: The walls, floors and interior of the sauna area were disinfected. The sauna cabins, sanitary facilities (toilets, showers, foot baths), and partition walls were foamed. Prior to disinfection, the surfaces were treated with an alkaline cleaning agent.

#19: Disinfection of a tiled area (415 m^2) next to the pool.

#20: In the sauna area, floor areas and corridors were disinfected as well as foot baths and the floors of the sauna itself. Prior to disinfection, the surfaces were treated with an alkaline cleaning agent.

Treated area #18 (sketch, not to scale):

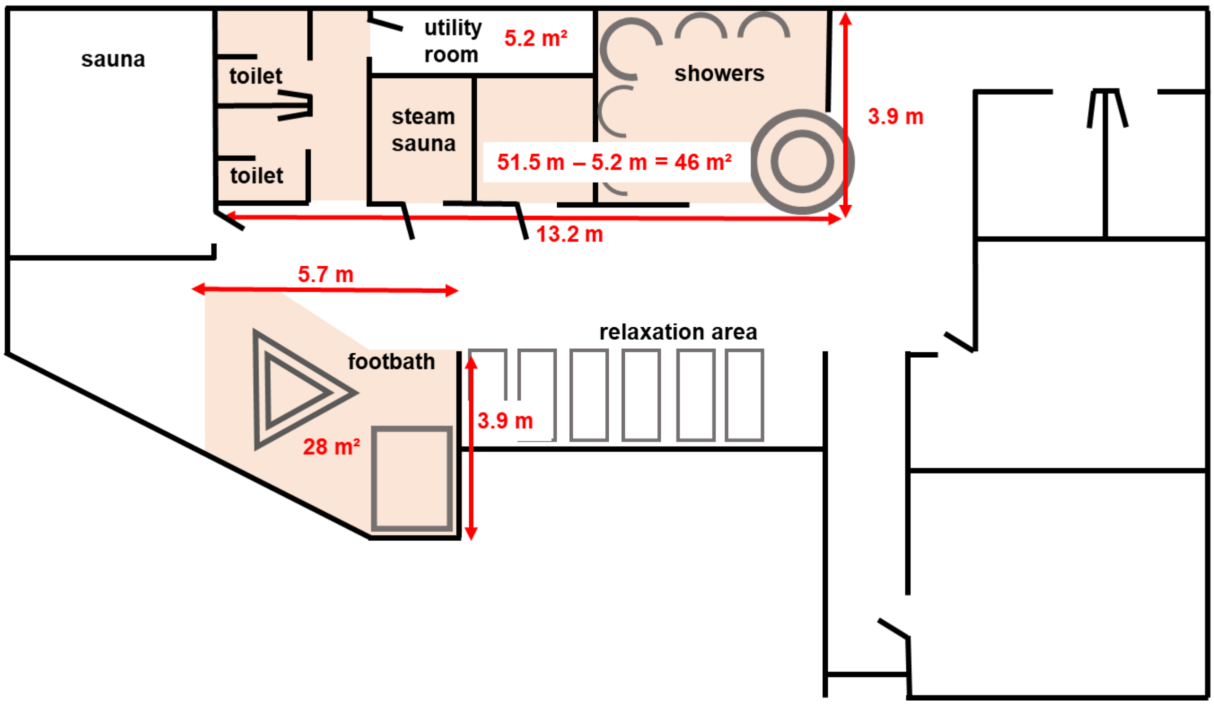

Room volume:	No information
Air-exchange rate:	No active air exchange
Room temp.:	26 °C
Rel. humidity:	63%

Treated area #19 (sketch, not to scale):

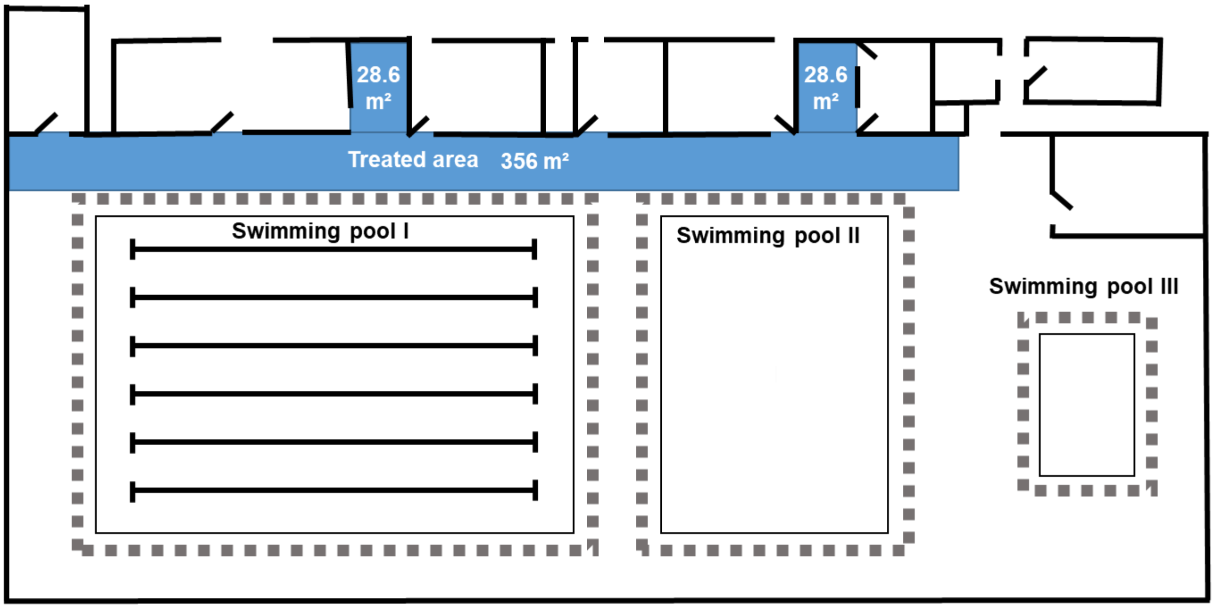

Room volume:	Approx. 10700 m³ (h: 7.45 m; l: 60 m; w: 20 m);
Air-exchange rate:	Max 35000 m³/h; depending on temperature and humidity, the air-exchange rate can be lowered to 50% of maximum capacity;
Room temp.:	32 °C
Rel. humidity:	64%

Treated area #20 (sketch, not to scale):

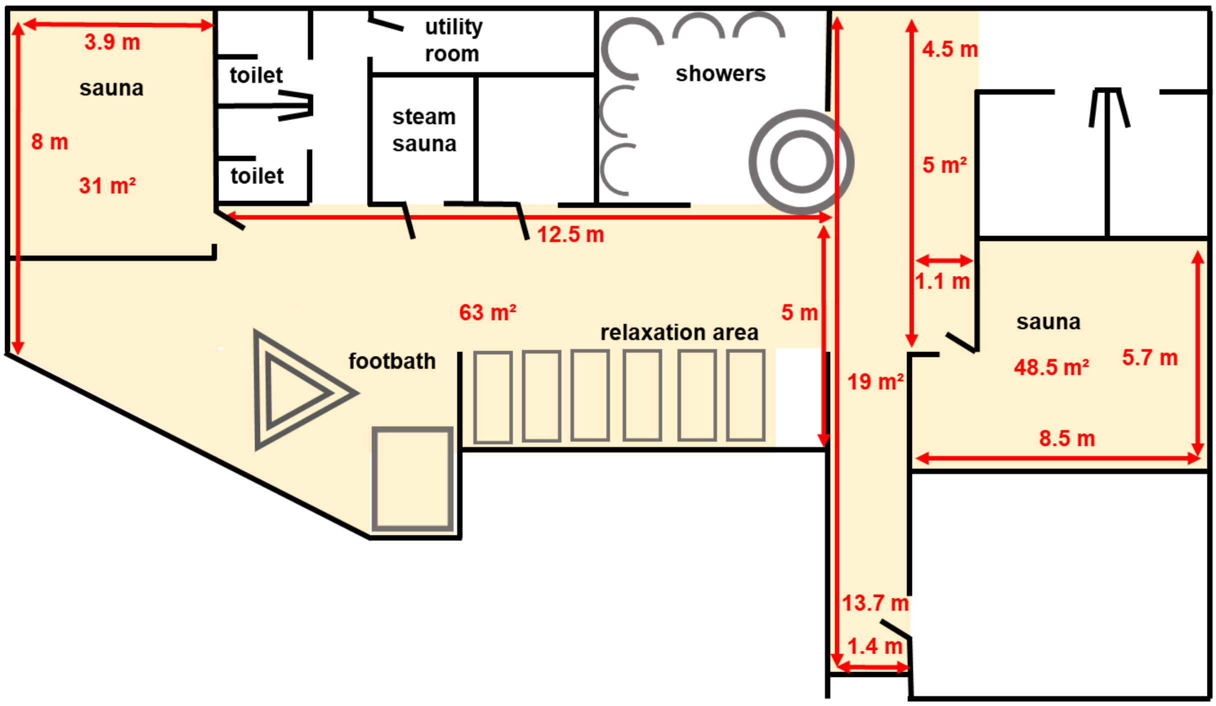

Room volume:	No information
Air-exchange rate:	No active air exchange
Room temp.:	29 °C
Rel. humidity:	61%

#	Application type	Device	Scenario description
#18 - F	Foam	Pressure foam gun with flat-fan nozzle, operated with a hose connected to a water tap (pressure of water mains: 3–5 bar)<br><br>Application solution was dosed to water jet according to the Venturi principle<br><br>Ratio foam-to-liquid volume: 3	Foam was rather liquid and applied in straightforward movements. Application was mostly performed in left-and-right vertical motions (floor and lower wall areas) and in up-and-down, left-and-right motions (upper wall areas and steam sauna), including overhead application (up to 2.7 m). Horizontal areas up to a height of 1.2 m were also treated. Given the complexity of the sauna area, the treated area is estimated to be approx. 140 m².<br>The distance between hand and nozzle was approx. 25 cm. The distance to the treated surface was approx. 0.5–1.2 m.<br><br>Treated area: 140 m²<br><br><u>Contact with biocidal product/treated surface</u>: The foam gun was held with the right hand and the water hose with the left hand. The operator closed a door in the shower area. Disinfectant dripped from the nozzle onto her right hand and arm as well as her feet and lower legs. The hood of the coverall was left off.<br><br>Application duration: 5.85 min
#19 - H	Spray	Battery-powered pressure sprayer<br><br>Pressure (device): 2 bar<br><br>Flat-fan nozzle M40040 (angle: 40°; capacity: 3.5 L/min)	Application was performed at a rate of approx. 2.4 L/min. The diluted biocidal product (application solution) was in the reservoir of the battery-powered pressure sprayer and was applied by a spraying lance. The spraying lance was held with the right hand and the battery-powered pressure sprayer was pulled behind with the left, whereby the leading arm was mostly outstretched. The disinfectant was applied in straightforward movements, whereby the distance from the nozzle to the treated surface was about 20–50 cm; the distance between spraying nozzle and hand was about 60 cm. The pool borders were disinfected with horizontal motions from right to left; the five benches at the side and both shower areas were disinfected using the same pendulum movement. The treated surfaces were located at the height of the legs (floor and lower wall area), and some stood at pelvic height (seatbacks of benches).<br><br>Treated area: 415 m²<br><br><u>Contact with biocidal product/treated surface</u>: The hands were in direct contact with the spray lance and battery-powered sprayer; water from the floor splashed back onto the lower legs.<br><br>Application duration: 16 min

#	Application type	Device	Scenario description
#20 - G	Foam	Pressure foam gun with flat-fan nozzle, operated with a hose connected to a water tap (pressure of water mains: 3–5 bar)<br><br>Application solution was dosed to water jet according to the Venturi principle<br><br>Ratio foam-to-liquid volume: 4	The foam lance was mainly held with the right hand and the water hose was pulled/held with the left, whereby the leading arm was outstretched. Foam was applied in straightforward movements and the floor was disinfected in horizontal movements. All treated areas were at leg level (floor and foot baths). The applied foam was rather liquid. The distance between hand and nozzle was approx. 25 cm. The distance to the treated surface was approx. 0.6–1.2m.<br><br>Treated area: approx. 167 m²<br><br>Contact with biocidal product/treated surface: The hands were in direct contact with the foam lance and water hose. The operator also came into contact with lounge chairs in the relaxation area when moving them to disinfect the floor underneath.<br>The operator removed the disinfectant solution from the floor using a telescopic squeegee (touching its handle). The operator attached the foam gun to the water hose and screwed the dispenser head onto the foam gun.<br><br>Application duration: 5.2 min

Application scenario: #21

Product type: Disinfectants

Biocidal product (BP): QAC E

Active substance (a.s.): 74 g/L Benzyl-(C12–C16)-alkyldimethylammoniumchloride (BAC)

46 g/L Didecyldimethylammonium chloride;

112 g/L Glutaral;

Application solution: 0.4 g BAC/L

Type: Disinfection of surfaces (large-scale)

Operator I: Professional user (right-handed) conducting QAC-based disinfections once every two weeks; height: 1.91 m; weight: 86 kg

Personal protective equipment for the operator: Cotton sampling gloves over blue nitrile gloves, Tyvek® coverall (sampling media) over rubber coverall, full-face respiratory protection, Wellington boots

Note: The belt, which was equipped with the pumps and sample collectors needed for aerosol measurements, covered small parts of the upper body.

Work procedure for #21

The biocide was applied by one person. A pigsty as part of an animal farm was disinfected using QAC-containing disinfectant. The disinfectant was applied with a pressure foam gun. The pressure foam gun was used to dose the biocidal product and foaming agent to the water jet during foam application. The floor, walls, and interiors of partitions/boxes were treated.

Treated area (sketch, not to scale):

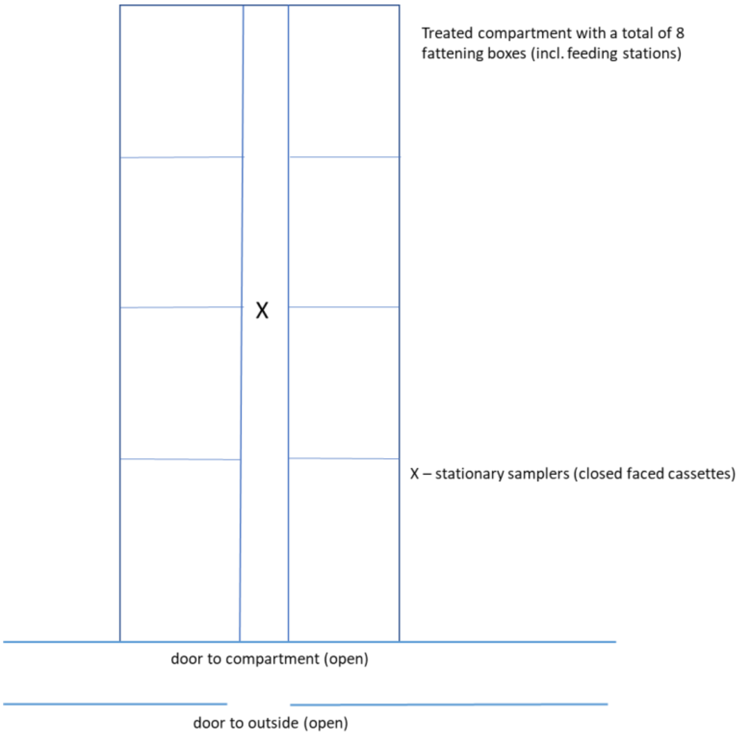

Room volume:	616 m³
Air-exchange rate:	No active air exchange; both doors were open
Room temp.:	17 °C
Rel. humidity:	47%

#	Application type	Device	Scenario description
#21 - I	Foam	Pressure foam gun with flat-fan nozzle, operated with a stationary high-pressure cleaner (device pressure: 150 bar)<br><br>Flat-fan nozzle<br><br>Ratio foam-to-liquid volume: 3 (probably too low as foam was directly injected into measuring cylinder)	The foam lance was mainly operated with the right hand. The left hand was placed on top of the lance to stabilise it. The right arm was held fairly close to the body at the level of the stomach and chest during foaming. The foam was thick and stable. Foam application was conducted in straightforward movements. At the end of application, the operator applied the foam in a backwards motion down the centre aisle. The treated surfaces were mainly at the height of the legs (floor and lower wall area) and stomach (partition walls of the fattening boxes) as well as at the height of the chest and overhead (upper wall area). The foam was applied in up-and-down motions along the high walls but in horizontal, back-and-forth motions on the floor and low partition walls of the boxes.<br>The distance between hand and nozzle was approx. 25 cm. The distance of the nozzle to the treated surface (floor and partitioning walls of boxes) was approx. 0.5–1 m or 2 m for the upper end of the stable walls.<br><br>Treated area: approx. 504 m²<br><br>Contact with biocidal product/treated surface: Both hands were in direct contact with the foam lance. Treated partitioning doors were opened and closed with the left hand. A measuring cylinder was used to transfer the application solution into the reservoir of the pressure foam gun. The left hand touched the measuring cylinder.<br><br>Application duration: 12 min

Application scenario: #24–#26

Product type: Mould, moss, and algae remover

Biocidal product (BP): QAC M

Active substance (a.s.): 95 g/kg Alkylbenzyldimethylammonium chloride (BAC)

Application solution: 15 g/L BAC;

0.1% CsCl (tracer, spiked)

Type: Treatment of surfaces (large-scale)

Operator D: Pest-control professional (right-handed) applying QAC-based biocidal products once a month; height: 1.67 m; weight: 66 kg

Personal protective equipment for operator: Cotton sampling gloves over blue nitrile gloves, Tyvek® coverall (sampling media), respiratory protection (FFP2 mask), work shoes, safety goggles

Note: The belt, which was equipped with the pumps and sample collectors needed for aerosol measurements, covered small parts of the upper body.

Work procedure for #24–#26

In each case, the biocide was applied by one person. The application solution was applied in a room of about 22 m². All walls (height: 2.49 m) were treated partially twice or twice, omitting windows and doors. The treated surfaces were at the height of the legs and stomach (lower wall areas) as well as overhead (upper wall areas). During application, the operator faced the respective wall. Application was performed mostly in horizontal back-and-forth movements for #24 and #26 and in both horizontal back-and-forth movements and vertical up-and-down movements for #25.

Treated area (sketch, not to scale):

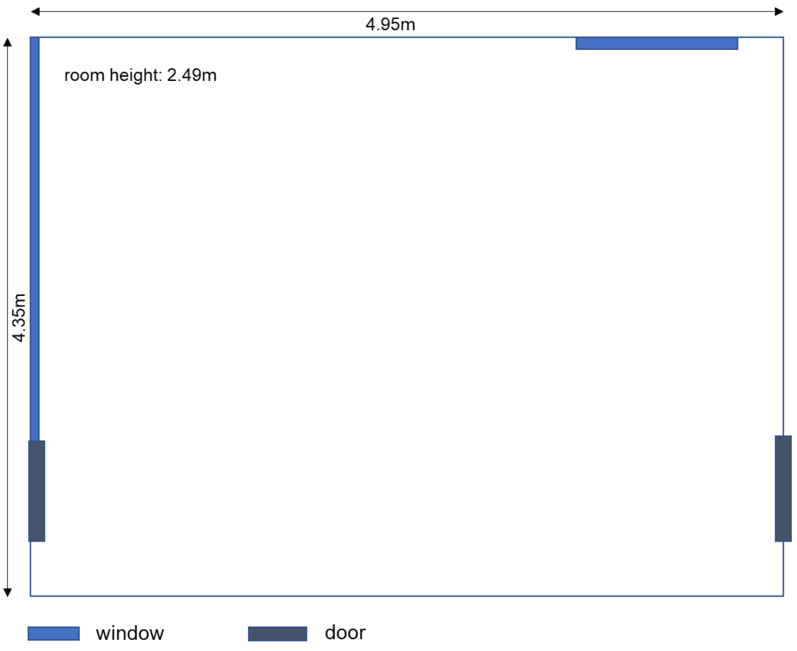

Room volume:	54 m³
Air-exchange rate:	Closed room, no active air exchange
Room temp.:	16–17 °C
Rel. humidity:	69–74%

#	Application type	Device	Scenario description
#24 - D	Foam	Pressure foamer P (1–3 bar); universal foam nozzle (75.4 mm² orifice)<br><br>Pressure (device): not controlled, estimated: 2–3 bar<br><br>Ratio foam-to-liquid volume: 5	Before the first application, the operator loaded the foamer wearing the Tyvek® coverall but no cotton gloves. The pressure foamer required multiple manual adjustments of device pressure both before and during application.<br>The walls were partially treated twice. The applied foam was rather liquid and dripped down the walls.<br>The foam lance was operated with the right hand, and the pressure foamer was carried with the left. During surface treatment at the bottom end of the walls, the right arm was bent and held closer to the body; when applying overhead, the arm holding the foam lance was extended. The distance between nozzle and treated surface was approx. 5 cm. The distance between nozzle and hand was approx. 40–45cm. The distance between operator and treated surface was approx. 50 cm.<br>Treated area: approx. 57 m²<br><br><u>Contact with biocidal product/treated surface</u>:<br>Foamer pressure was mostly adjusted with the left hand. The nozzle was readjusted once, also using the left hand. During the work procedure, the reservoir had to be refilled; this step was performed by an assistant, not the operator himself. For this purpose, the door had to be opened for a brief moment. The operator, however, took the canister of application solution in both hands. While refilling the reservoir, the operator knelt on the floor and held the foam lance in his left hand. After the second refill, the operator screwed the foamer shut using his left hand, using his right hand to prop himself up on his right thigh. During foam application, the operator carried the foamer with his left hand, whereby the foamer came into repeated contact with his left leg.<br><br>Application duration: 20 min
#25 - D	Foam	Pressure foamer P (1–3 bar), flat-fan nozzle (2.75 mm² orifice) in conjunction with a foam cartridge (highly foaming)<br><br>Pressure (device):<br>2.4 bar, the required pressure for the foamer was kept at a constant level using an external compressor.<br><br>Ratio foam-to-liquid volume: 10.4	The foam was firmer than that of #24. The foam lance was operated with the right hand, and the pressure foamer was carried with the left. During surface treatment at the bottom end of the walls, the right arm was bent and held closer to the body; when applying overhead, the arm holding the foam lance was extended. Each wall was treated twice.<br>The distance between nozzle and treated surface was approx. 30 cm. The distance between nozzle and hand was approx. 45 cm. The distance between operator and treated surface was approx. 100 cm.<br>Treated area: approx. 67 m²<br><br><u>Contact with biocidal product/treated surface</u>: The operator took the pressure foamer in both hands and unscrewed the lid to refill the reservoir with application solution. During the second refill, the operator knelt on the floor with both knees. He also knelt down briefly to treat the floor strips. Foam application performed overhead produced tiny foam flakes which settled on the operator. During application, the operator adjusted his safety goggles twice and touched his head/hood several times with his left hand.<br><br>Application duration: 31 min

#	Application type	Device	Scenario description
#26 - D	Spray	Pressure sprayer P (1–3 bar); hollow cone nozzle<br><br>Pressure (device): not controlled, estimated: 2–3 bar	The pressure sprayer required multiple manual adjustments of device pressure both before and during application. The spray lance was operated with the right hand, and the pressure sprayer was carried with the left. During application, the arm holding the spray lance was extended. Three of the four walls were treated twice. The distance between nozzle and treated surface was approx. 20–25 cm. The distance between nozzle and hand was approx. 40–45cm. The distance between operator and treated surface was approx. 80–100 cm.<br><br>Treated area: approx. 59 m²<br><br><u>Contact with biocidal product/treated surface</u>:<br>Sprayer pressure was mostly adjusted with the left hand. The nozzle was readjusted once, also using the left hand. During application, the operator moved flowerpots on the windowsill.<br><br>Towards the end of application, the operator placed the sprayer on his left thigh, tilting it to apply the remaining amount of application solution.<br><br>Application duration: 12 min

Sampling procedures

Air monitoring

To determine inhalable aerosol exposure during spray or foam application, personal sampling was conducted using GSP sampling heads (samplers for inhalable aerosols, ANALYT-MTC Meßtechnik GmbH, Müllheim, Germany) at flow rates of either 3.5 mL/min or 10 mL/min. Two GSP samplers were positioned within the breathing zone of the operator. Depending on the analyte, active substance, or tracer selected for the determination of inhalable aerosol exposure, a suitable 37 mm filter was chosen for sample collection: MCE (mixed cellulose ester) filters for CsCl; PTFE (polytetrafluoroethylene)-coated filters for BAC, and glass-fibre filters for pyrethroids.

Monitoring of dermal exposure

Sampling procedures and analytical methods were developed in consideration of OECD guidance document No. 9 (OECD/D(97)148) (OECD 2017). Potential dermal exposure was quantified using whole-body dosimeters comprised of whole-body coveralls with hoods made of high-density polyethylene (DuPont® Tyvek® Classic Expert CHF5 protective coverall cat. III; type 5B and 6B) and cotton gloves. Study personnel assisted the workers in putting on the dermal samplers to avoid cross-contamination. Coveralls and gloves were worn throughout spray and foam applications and were removed at the end of each application. Following sampling, the dosimeters were either cut into 11 segments (coveralls) or left whole (gloves) (Supplemental Figure 2). Each sample was wrapped in aluminium foil and transported back to the lab at 4 °C, where they were stored at −20 °C until analysis. Dosimeter workup and extract analysis were performed within six weeks of the field study. Individual extracts had to be diluted or concentrated, then newly analysed within ten weeks.

Determination of non-volatile active substances and tracer in air and on dermal samplers

Inhalation exposure

Pyrethroids

Sample analysis was based on VDI Guideline 4301, Part 4 (VDI/DIN 2016). Briefly, filters were cut into pieces, transferred into a beaker, and, after addition of the internal standard (piperonyl butoxide-d_9), extracted with ethyl acetate in an ultrasonic bath in three successive steps. The combined supernatant was reduced to a volume of 0.5 mL under a nitrogen stream, then made up to a final volume of 1 mL with ethyl acetate. Quantification of permethrin and phenothrin was achieved by gas chromatography with mass-spectrometric detection (GC-MS). As for piperonyl butoxide (PBO), the analysis of permethrin is described in VDI Guideline 4301, Part 4. The successful implementation of this method was demonstrated with PBO. This method should therefore also be suitable for the quantification of permethrin, as was confirmed by a plausibility check using measurement data; the ratio between permethrin and PBO in the filter extracts was equal to that of the application solution. During method validation, including permethrin was not yet known to be necessary.

During a method-validation procedure conducted in the same laboratory (Elflein et al. 2003), theoretical quantitation limits of 1 ng permethrin/m^3 and 0.3 ng PBO/m^3 were calculated, and a practical limit of quantification (lowest calibration standard) of 10 ng/m^3 was applied. In both instances, a sample volume of 10 m^3 was assumed. In this study, the limit of quantification for PBO could be lowered by a factor of about 3, most likely due to technological advancement.

Analyte stability (namely: PBO, tetramethrin, and phenothrin) on filter material at 7–12 °C and at room temperature was investigated during method validation. Stability could be demonstrated for a time period of at least 8 days and 24 h, respectively. For permethrin, VDI Guideline 4301 confirms stability for approximately two weeks when filters are stored at room temperature and in the dark. Samples were analysed within this timeframe.

**Theoretical limit of quantification:**<br>(noise + 10x standard deviation of noise)	PBO: 0.005 µg/m³ (for an air sample volume of 0.21 m³ and a liquid sample volume of 1 mL)<br><br>Tetramethrin: 0.005 and 0.02 µg/m³ (Peak 2 and 1); (for an air sample volume of 0.21 m³ and a liquid sample volume of 1 mL)<br><br>Phenothrin: 0.02 µg/m³ (for an air sample volume of 0.21 m³ and a liquid sample volume of 1 mL)
**Practical limit of quantification:**<br>(lowest calibration standard)	0.024 µg/m³ (for an air sample volume of 0.21 m³ and a liquid sample volume of 1 mL)<br><br>5 ng/mL (sample volume (liq): 1 mL)
**Accuracy:**	
PBO	91.4–115%
Tetramethrin	82.1–112%
Phenothrin	82.6–105%
**Precision:**	
PBO	rel. standard deviation: 1.3–13.4%
Tetramethrin	rel. standard deviation: 2.2–14.1%
Phenothrin	rel. standard deviation: 3.7–15.8%
**Estimated expanded uncertainty**:**	
PBO	32%
Tetramethrin	34%
Phenothrin	34%

*Duration of sampling: 60 min at 3.5 L/min (sampling with GSP)
** Calculation according to IFA; URL: http://www.dguv.de/ifa/praxishilfen/praxishilfen-gefahrstoffe/software-berechnung-der-erweiterten-messunsicherheit-nach-ifa/index.jsp

Benzalkonium chlorides (BAC)

Sample analysis was based on the work of Van Boxtel et al (2016). Briefly, after sampling and spiking the filter with the internal standard (BAC-d_7), the filters were transferred into headspace vials and the residual solvent was removed under a nitrogen stream. After thermal degradation of BAC, the resulting benzyl chloride was sampled from the headspace and quantified by GC-MS analysis. During validation, the storage stability for benzalkonium chloride on PTFE filters was investigated at 7–12 °C and at room temperature and was confirmed over a period of at least two weeks. Samples were analysed within the validated timeframe.

**Theoretical limit of quantification:** (noise + 10x standard deviation of noise)	0.0095 µg/m³ (for an air sample volume* of 0.21 m³)
**Theoretical limit of quantification:** (lowest calibration standard)	2.4 µg/m³ (for an air sample volume* of 0.21 m³) 0.5 µg/filter
**Accuracy:**	95.2–113%[#]
**Precision:**	2.5–22.8%

For the measurement range of 0.5–6 µg/filter (2.4–29 µg/m³); at a sample volume* of 0.21 m³.

**Estimated expanded uncertainty**:**	35%

*Duration of sampling: 60 min at 3.5 L/min (sampling with GSP)
#Obvious outliers were omitted
**Calculation according to IFA; URL: http://www.dguv.de/ifa/praxishilfen/praxishilfen-gefahrstoffe/software-berechnung-der-erweiterten-messunsicherheit-nach-ifa/index.jsp

Cesium chloride (CsCl)

Filter samples were extracted with 25 mL of 0.15% hydrochloric acid in 50 mL polypropylene tubes using an overhead shaker. Aliquots were either diluted or analysed for their Cs content directly after adding the internal standards, lutetium and indium, by inductively coupled plasma-mass spectrometry (ICP-MS).

**Theoretical limit of quantification:** (according to* DIN 32645)	0.025 µg/m³ (for an air sample volume** of 0.21 m³ and a liquid sample volume of 25 mL)
	0.21 ng/mL (extraction volume: 25 mL)
**Practical limit of quantification:** (lowest calibration standard)	0.075 µg/m³ (for an air sample volume** of 0.21 m³ and a liquid sample volume of 25 mL)
	0.5 ng/mL (sample volume: 50 mL); 25 ng/filter
**Recovery:**	99.6%, concentration 0.15 µg/m³ (n=6; 1 ng/mL in filter extract)
	97.6%, concentration 3 µg/m³ (n=6; 1 ng/mL in filter extract)
**Precision:**	rel. standard deviation <1% for a concentration range of 0.15–3 µg/m³ (n=6)
**Estimated expanded uncertainty***:**	20%

* For the calibration range between 0.075 µg/m³ and 3 µg/m³ (0.5 ng/mL and 20 ng/mL in aqueous solution)
** Duration of sampling: 60 min at 3.5 L/min (sampling with GSP)
*** Calculation according to IFA; URL: http://www.dguv.de/ifa/praxishilfen/praxishilfen-gefahrstoffe/software-berechnung-der-erweiterten-messunsicherheit-nach-ifa/index.jsp

Dermal exposure

Pyrethroids

Sampler material was extracted by shaking the coverall segments and gloves with acetone for five minutes each. The solvent volume was adjusted according to sample size. A 1-mL aliquot of the extract was used for further processing. After the addition of bifenthrin as an internal standard, the sample was evaporated to dryness under a stream of nitrogen and the residue was resuspended in 1 mL of toluene. The sample was subsequently filtered using a 1-µm syringe filter, and the pyrethroid levels were quantified by GC-MS.

**Theoretical limit of quantification (according to DIN 32645):**	
Tetramethrin	1.69 µg/L
trans-Phenothrin	1.23 µg/L
Permethrin	1.60 µg/L
Tyvek® material:	0.07–0.1 ng/cm² for tetramethrin, *trans*-phenothrin and permethrin
Cotton gloves:	0.2–0.3 ng/cm² for tetramethrin, *trans*-phenothrin and permethrin
**Accuracy:**	
Tetramethrin	91.4–104% (Tyvek®); 85.7–97.0% (cotton)
trans-Phenothrin	91.4–113% (Tyvek®); 87.6–99.4% (cotton)
Permethrin	91.3–110% (Tyvek®); 88.8–106% (cotton)
**Day-to-day precision:**	
Tetramethrin	5.97–8.38%
trans-Phenothrin	5.11–9.62%
Permethrin	3.13–6.51%

For the measurement range of 5–50 µg/L (Tyvek® material) and 2.5–25 µg/L (cotton) (accuracy, n=3); 10–500 µg/L (precision, n=8)

Quality-control material spiked at three levels, as well as reagent and dosimeter blanks, were included in all analytical runs. No analyte blanks were found in the reagents or dosimeter materials. The quality-control charts did not show any discrepancies over time.

Storage stability of the analytes was tested by spiking 900 cm² Tyvek® material or one cotton glove with 0.25 µg or 2.5 µg of the pyrethroids, respectively (n=3). The sampler material was wrapped in aluminium foil, sealed in polyethylene bags, and stored for 60 days at −20 °C and 6 °C. No storage-related analyte losses were detectable. The mean recovery rates lied

between 85.7% and 106%. Samples were analysed within the validated timeframes. Field recovery was not conducted in parallel to the field study.

Benzalkonium chlorides (BAC)

Sampler material was extracted by shaking the coverall segments and gloves with acetone for five minutes each. The solvent volume was adjusted according to sample size. A 1-mL aliquot of the extract was pipetted into a headspace vial and used for further processing. After the addition of the deuterated internal standard, the solvent was removed under a stream of nitrogen, the vial was capped (high-temperature septa), and the residue was heated to 170 °C for thermal decomposition of the analytes. Benzyl chloride, the thermal decomposition product of BAC, was quantified using headspace-GC-MS.

**Limit of quantification (according to DIN 32645):**	0.019 µg/vial (2 µg/L)
Tyvek® material:	1.1 ng/cm² (50 mL extraction liquid for 900 cm²)
Glove:	3.3 ng/cm² (100 mL extraction liquid for one glove)
**Accuracy:**	90.0–102% (Tyvek®) for the amount 0.12–0.6 µg BAC/vial (n=3);<br>95.9–105% (cotton) for the amount 0.06–0.3 µg BAC/vial (n=3)
**Precision:**	2.62–6.12% for the amount of 0.1–5 µg BAC/vial (n=8)

For the measurement range of 5–50 µg/L (Tyvek® material) and 2.5–25 µg/L (cotton) (accuracy, n=3);10–500 µg/L (precision, n=8)

Quality-control material spiked at three levels, as well as reagent and dosimeter blanks, were included in all analytical runs. No analyte blanks were found in the reagents or dosimeter materials. The quality-control charts did not show any discrepancies over time.

Storage stability of the analytes was tested by spiking 900 cm² Tyvek® material or one cotton glove with 6 µg or 30 µg BAC, respectively (n=3). The sampler material was wrapped in aluminium foil, sealed in polyethylene bags, and stored for 60 days at −20°C and 6°C. No storage-related analyte losses were detectable. The mean recovery rates lied between 94.7%

and 107%. Samples were analysed within the validated timeframes. Field recovery was not conducted in parallel to the field study.

Application solution

The concentration of active substance in the application solution and, where applicable, tracer concentration, were determined after an appropriate dilution step employing the same analytical methods as for the determination of inhalation and dermal exposure.

References

Elflein, L., Berger-Preiss, E., Levsen, K., Wünsch, G. (2003) Development of a gas chromatography-mass spectrometry method for the determination of household insecticides in indoor air. J Chromatogr A, 985, 147–157. Available from: DOI: 10.1016/S0021-9673(02)01461-9

OECD (2017): Environmental Health and Safety Publications. Series on Testing and Assessment. No. 9: Guidance Document for the Conduct of Studies of Occupational Exposure to Pesticides During Agricultural Application (OECD/GD(97)148). Paris: OECD 1997

VDI/DIN (2016) Guideline 4301, Part 4. Measurement of indoor air pollution – Measurement of pyrethroids and piperonyl butoxide in air. In: Handbuch Reinhaltung der Luft.

van Boxtel N, Wolfs K, Palacín MG, Van Schepdael A, Adams E. (2016) Headspace gas chromatography based methodology for the analysis of aromatic substituted quaternary ammonium salts. J Chromatogr A; 1476: 105–113. Available from: DOI: 10.1016/j.chroma.2016.11.009

Supplementary material – Tables

Tab. S1 Active substance quantified on the coverall segments; absolute amounts of active substance [µg] (n = 26).

#	Active substance	Exposure of body areas [µg]											
		Breast/ Stomach	Back/ Buttocks	Upper arm, right	Upper arm, left	Forearm, right	Forearm, left	Thigh, right	Thigh, left	Lower leg, right	Lower leg, left	Hood	Sum
**1 - A**	BAC*	131	2.35	48.2	0.379	1810	7.24	2.38	11.7	0.391	0.455	0.561	**2020**
**2 - A**	BAC*	24.6	0.762	2.32	0.457	289	3.20	0.305	0.409	0.308	0.482	0.343	**322**
**3 - A**	BAC*	33.0	1.41	19.1	0.261	760	6.73	0.475	0.451	0.943	2.41	1.03	**826**
**4 - A**	BAC*	22.7	3.09	37.2	0.498	253	4.70	1.37	0.786	1.00	1.04	1.27	**327**
**5 - C**	BAC*	9.67	9.78	2.26	3.19	0.759	1.84	4.96	3.99	8.50	9.89	0.768	**55,6**
**6 - C**	BAC*	11.5	12.7	5.10	8.39	1.64	1.82	8.15	5.64	35.5	34.3	2.31	**127**
**7 - C**	BAC*	17.6	15.7	16.4	10.3	47.2	3.94	24.6	33.3	112	60.7	42.1	**383**
**8 - C**	BAC*	5.16	1.81	1.86	1.64	1.26	0.660	3.34	2.10	8.08	7.39	0.936	**34,2**
**9 - B**	BAC*	17.8	5.90	2.48	2.85	3.53	10.2	5.54	2.91	2.20	2.28	1.79	**57,5**
**10 - B**	BAC*	24.4	19.3	3.90	4.06	4.82	205	12.6	10.7	20.2	16.1	6.33	**327**
**11 - C**	Phenothrin	0.377	0.078	0.089	0.112	0.787	0.100	0.034	0.076	0.029	0.057	0.040	**1,78**
**12 - C**	Phenothrin	0.258	0.060	0.120	0.061	80.3	0.201	0.021	0.199	0.011	0.106	0.010	**81,4**
**13 - C**	Phenothrin	36.3	49.3	33.1	30.7	48.5	68.5	6.37	7.60	11.3	7.24	57.1	**356**
**14 - E**	BAC*	266	50.5	21.5	62.5	58.5	47.7	139	176	53.3	84.4	42.4	**1000**
**15 - E**	BAC*	453	69.5	31.6	32.3	58.7	56.9	95.7	115	159	224	71.5	**1370**
**16 - E**	BAC*	278	210	89.7	119	94.4	64.6	151	189	215	255	148	**1810**
**17 - E**	BAC*	1440	1420	806	307	1030	400	714	784	1770	1150	936	**10800**
**18 - F**	BAC*	46.9	15.0	5.86	12.9	8.55	77.1	60.9	76.8	237	210	1.02	**752**
**19 - H**	BAC*	60.2	35.8	5.51	3.65	15.8	7.47	49.7	54.3	243	156	4.64	**637**
**20 - G**	BAC*	20.6	13.1	5.44	3.14	5.26	5.16	9.72	39.5	66.0	155	2.48	**325**
**21 - I**	BAC*	2100	58.8	7.94	5.35	7.02	206	900	818	1160	1900	20.3	**7190**
**24 - D**	BAC*	4400	68.3	32.9	5.08	309	23.6	1400	8060	679	713	43.2	**15700**
**25 - D**	BAC*	414	344	374	90.8	498	111	144	215	190	234	218	**2830**
**26 - D**	BAC*	8370	371	130	94.1	140	124	204	4360	421	389	231	**14800**
**27 - D**	Phenothrin	8.14	2.98	0.426	364	6.04	205	0.492	10.9	0.885	1.40	0.907	**600**
**28 - D**	Permethrin	21.2	3.20	5.74	1.40	79.6	8.67	11.9	2.25	1.15	9.19	2.31	**147**

* BAC = Benzalkonium chlorides

Tab. S2 Active substance quantified on the coverall segments; absolute amounts of active substance normalised to the amount of active substance applied during the task [mg/kg] (n = 26).

#	Active substance	Exposure of body areas [mg/kg]											
		**Breast/ Stomach**	**Back/ Buttocks**	**Upper arm, right**	**Upper arm, left**	**Forearm, right**	**Forearm, left**	**Thigh, right**	**Thigh, left**	**Lower leg, right**	**Lower leg, left**	**Hood**	**Sum**
**1 - A**	BAC*	252	4.53	92.7	0.729	3490	13.9	4.58	22.6	0.752	0.875	1.08	**3880**
**2 - A**	BAC*	244	7.55	23.0	4.53	2860	31.7	3.03	4.06	3.05	4.78	3.40	**3190**
**3 - A**	BAC*	216	9.22	125	1.71	4970	44.0	3.10	2.95	6.17	15.8	6.73	**5400**
**4 - A**	BAC*	210	28.5	343	4.59	2330	43.3	12.7	7.24	9.25	9.55	11.7	**3010**
**5 - C**	BAC*	0.581	0.588	0.136	0.191	0.046	0.110	0.298	0.240	0.511	0.594	0.046	**3.34**
**6 - C**	BAC*	2.47	2.73	1.10	1.81	0.354	0.392	1.76	1.22	7.66	7.40	0.498	**27.4**
**7 - C**	BAC*	0.918	0.816	0.854	0.535	2.46	0.205	1.28	1.74	5.81	3.16	2.19	**20.0**
**8 - C**	BAC*	4.03	1.41	1.45	1.28	0.981	0.516	2.61	1.64	6.31	5.78	0.732	**26.7**
**9 - B**	BAC*	19.9	6.60	2.78	3.18	3.94	11.4	6.19	3.25	2.46	2.55	2.00	**64.2**
**10 - B**	BAC*	29.4	23.2	4.70	4.89	5.81	247	15.2	12.9	24.4	19.4	7.63	**394**
**11 - C**	Phenothrin	0.457	0.095	0.108	0.136	0.955	0.121	0.042	0.092	0.035	0.069	0.048	**2.16**
**12 - C**	Phenothrin	0.463	0.107	0.215	0.110	144	0.360	0.037	0.357	0.019	0.191	0.018	**146**
**13 - C**	Phenothrin	109	148	99.1	91.9	145	205	19.1	22.8	34.0	21.7	171	**1070**
**14 - E**	BAC*	53.0	10.1	4.29	12.5	11.7	9.50	27.8	35.2	10.6	16.8	8.45	**200**
**15 - E**	BAC*	3.94	0.604	0.275	0.281	0.510	0.495	0.832	1.00	1.38	1.95	0.622	**11.9**
**16 - E**	BAC*	2.06	1.55	0.665	0.878	0.699	0.478	1.12	1.40	1.59	1.89	1.10	**13.4**
**17 - E**	BAC*	10.9	10.8	6.11	2.33	7.78	3.03	5.41	5.94	13.4	8.74	7.09	**81.5**
**18 - F**	BAC*	4.11	1.32	0.514	1.14	0.750	6.76	5.34	6.74	20.8	18.4	0.090	**66.0**
**19 - H**	BAC*	0.930	0.552	0.085	0.056	0.245	0.115	0.767	0.838	3.76	2.42	0.072	**9.83**
**20 - G**	BAC*	1.81	1.15	0.477	0.276	0.461	0.453	0.853	3.46	5.79	13.6	0.217	**28.5**
**21 - I**	BAC*	14.2	0.397	0.054	0.036	0.047	1.39	6.08	5.53	7.82	12.9	0.137	**48.6**
**24 - D**	BAC*	23.3	0.362	0.174	0.027	1.64	0.125	7.40	42.7	3.60	3.78	0.229	**83.4**
**25 - D**	BAC*	2.60	2.16	2.34	0.570	3.12	0.697	0.903	1.35	1.19	1.47	1.37	**17.8**
**26 - D**	BAC*	84.2	3.73	1.30	0.947	1.41	1.25	2.05	43.9	4.24	3.91	2.33	**149**
**27 - D**	Phenothrin	11.4	4.18	0.597	510	8.47	287	0.691	15.2	1.24	1.97	1.27	**842**
**28 - D**	Permethrin	3.08	0.465	0.834	0.203	11.6	1.26	1.73	0.327	0.167	1.33	0.336	**21.3**

* BAC = Benzalkonium chlorides

Tab. S3 Active substance quantified on the coverall segments; absolute amounts of active substance normalised to the amount of active substance applied during the task and to the segment area [µg/(kg×cm²)] (n = 26).

#	Active substance	Exposure of body areas [µg/(kg×cm²)]											
		**Breast/ Stomach**	**Back/ Buttocks**	**Upper arm, right**	**Upper arm, left**	**Forearm, right**	**Forearm, left**	**Thigh, right**	**Thigh, left**	**Lower leg, right**	**Lower leg, left**	**Hood**	**Mean**
**1 - A**	BAC*	37.0	0.685	57.5	0.452	2960	11.8	1.82	8.97	0.357	0.415	0.527	**128**
**2 - A**	BAC*	35.9	1.14	14.3	2.81	2430	26.9	1.20	1.61	1.45	2.27	1.66	**105**
**3 - A**	BAC*	31.8	1.39	77.5	1.06	4220	37.4	1.23	1.17	2.93	7.48	3.28	**178**
**4 - A**	BAC*	30.8	4.31	213	2.85	1980	36.8	5.03	2.88	4.39	4.53	5.72	**99.5**
**5 - C**	BAC*	0.085	0.089	0.084	0.119	0.039	0.094	0.119	0.095	0.242	0.282	0.023	**0.110**
**6 - C**	BAC*	0.363	0.413	0.682	1.12	0.301	0.333	0.698	0.483	3.64	3.51	0.243	**0.904**
**7 - C**	BAC*	0.135	0.123	0.530	0.332	2.09	0.175	0.509	0.690	2.76	1.50	1.07	**0.659**
**8 - C**	BAC*	0.592	0.213	0.900	0.793	0.833	0.438	1.04	0.653	2.99	2.74	0.357	**0.883**
**9 - B**	BAC*	2.93	1.00	1.72	1.97	3.35	9.67	2.46	1.29	1.17	1.21	0.976	**2.12**
**10 - B**	BAC*	4.32	3.51	2.92	3.03	4.94	210	6.05	5.14	11.6	9.23	3.72	**13.0**
**11 - C**	Phenothrin	0.067	0.014	0.067	0.084	0.811	0.103	0.017	0.037	0.017	0.033	0.023	**0.071**
**12 - C**	Phenothrin	0.068	0.016	0.133	0.068	122	0.306	0.015	0.142	0.009	0.091	0.009	**4.82**
**13 - C**	Phenothrin	16.0	22.3	61.5	57.0	123	174	7.58	9.05	16.1	10.3	83.4	**35.2**
**14 - E**	BAC*	7.79	1.52	2.66	7.73	9.90	8.07	11.0	14.0	5.05	7.99	4.12	**6.60**
**15 - E**	BAC*	0.579	0.091	0.171	0.174	0.433	0.420	0.331	0.398	0.656	0.925	0.303	**0.393**
**16 - E**	BAC*	0.303	0.235	0.412	0.545	0.594	0.406	0.445	0.557	0.756	0.898	0.535	**0.444**
**17 - E**	BAC*	1.61	1.63	3.79	1.44	6.61	2.57	2.15	2.36	6.38	4.15	3.46	**2.69**
**18 - F**	BAC*	0.604	0.200	0.319	0.704	0.637	5.74	2.12	2.68	9.89	8.73	0.044	**2.18**
**19 - H**	BAC*	0.137	0.084	0.053	0.035	0.208	0.098	0.305	0.333	1.78	1.15	0.035	**0.325**
**20 - G**	BAC*	0.266	0.174	0.296	0.171	0.392	0.385	0.339	1.38	2.75	6.43	0.106	**0.941**
**21 - I**	BAC*	2.09	0.060	0.033	0.022	0.040	1.18	2.42	2.20	3.71	6.11	0.067	**1.60**
**24 - D**	BAC*	3.43	0.055	0.108	0.017	1.39	0.106	2.94	17.0	1.71	1.80	0.112	**2.75**
**25 - D**	BAC*	0.382	0.327	1.45	0.353	2.65	0.592	0.359	0.536	0.567	0.698	0.666	**0.587**
**26 - D**	BAC*	12.4	0.565	0.809	0.588	1.20	1.06	0.816	17.4	2.01	1.86	1.14	**4.93**
**27 - D**	Phenothrin	1.68	0.632	0.370	316	7.20	244	0.275	6.05	0.589	0.935	0.620	**27.8**
**28 - D**	Permethrin	0.453	0.070	0.517	0.126	9.82	1.07	0.687	0.130	0.079	0.633	0.164	**0.703**

* BAC = Benzalkonium chlorides

Tab. S4 Active substance quantified on the gloves (n°=°30).

# (Gloves)	Active substance	Absolute exposure [µg]			Exposure normalised to the amount of active substance applied [mg/kg]			Exposure normalised to the amount of active substance applied and to the relevant segment area of 410 cm^2 per hand [µg/(kg×cm²)]		
		Right	Left	Total	Right	Left	Total	Right	Left	Mean
**1-1 - A (application)**	BAC*	<LOQ	9.35	10.3	<LOQ	18.0	19.9	<LOQ	43.9	24.3
**1-2 - A (wiping)**	BAC*	52800	<LOQ	52800	102000	<LOQ	102000	247800	<LOQ	124000
**1 - A (application + wiping)**	BAC*	52800	10.5	52800	102000	20.1	102000	247800	49.1	124000
**2-1 - A (application)**	BAC*	7.62	32.1	39.7	75.6	318	394	184	777	480
**2-2 - A (wiping)**	BAC*	7860	6.32	7870	78000	62.7	78000	190000	153	95200
**2 - A (application + wiping)**	BAC*	7870	38.4	7900	78000	381	78400	190000	930	95600
**3-1 - A (application)**	BAC*	6.42	<LOQ	7.62	42.0	<LOQ	49.8	102	<LOQ	60.7
**3-2 - A (wiping)**	BAC*	12600	113	12700	82600	740	83300	201000	1800	102000
**3 - A (application + wiping)**	BAC*	12600	114	12800	82600	748	83400	202000	1820	102000
**4-1 - A (application)**	BAC*	48.1	<LOQ	48.7	443	<LOQ	449	1080	<LOQ	548
**4-2 - A (wiping)**	BAC*	5440	60.2	5500	50100	555	50700	122000	1350	61800
**4 - A (application + wiping)**	BAC*	5490	60.9	5500	50600	561	51100	123000	1370	62400
**5 -C (application)**	BAC*	169	38.5	207	10.1	2.31	12.5	24.7	5.65	15.2
**6 - C (application)**	BAC*	14.4	34.9	49.3	3.10	7.52	10.6	7.56	18.3	13.0
**7 - C (application)**	BAC*	256	73.7	330	13.3	3.84	17.2	32.5	9.36	20.9
**8 - C (application)**	BAC*	12.4	9.91	22.3	9.66	7.74	17.4	23.6	18.9	21.2
**9 - B (application+ wiping)**	BAC*	14700	9660	24400	16500	10800	27300	40200	26300	33300
**10 - B (application + wiping)**	BAC*	4960	7530	12500	5980	9080	15100	14600	22100	18400
**11 - C (application)**	Phenothrin	180	32.9	213	218	39.9	258	533	97.4	315
**12 - C (application)**	Phenothrin	0.154	0.141	0.295	0.276	0.253	0.529	0.674	0.618	0.646

# (Gloves)	Active substance	Absolute exposure [µg]			Exposure normalised to the amount of active substance applied [mg/kg]			Exposure normalised to the amount of active substance applied and to the relevant segment area of 410 cm^2 per hand [µg/(kg×cm²)]		
		Right	Left	Total	Right	Left	Total	Right	Left	Mean
**13 - C (application)**	Phenothrin	21.0	50.9	71.8	62.8	152	215	153	372	262
**14 – E (application)**	BAC*	297	883	1180	59.2	176	235	144	429	287
**15 - E (application)**	BAC*	49.2	248	297	0.428	2.16	2.59	1.04	5.27	3.15
**16 - E (application)**	BAC*	60.6	35.4	95.9	0.449	0.262	0.711	1.09	0.639	0.867
**17 - E (application)**	BAC*	762	676	1440	5.77	5.12	10.9	14.1	12.5	13.3
**18 - F (application)**	BAC*	72.1	941	1010	6.32	82.6	88.9	15.4	201	108
**19 - H (application)**	BAC*	8.44	3.99	12.4	0.130	0.062	0.192	0.318	0.150	0.234
**20 - G (application)**	BAC*	30.0	275	305	2.63	24.1	26.7	6.41	58.8	32.6
**21 - I (application)**	BAC*	391	1070	1460	2.64	7.20	9.84	6.44	17.6	12.0
**24 - D (application)**	BAC*	67100	1530	68700	356	8.13	364	868	19.8	444
**25 - D (application)**	BAC*	508	1380	1890	3.19	8.64	11.8	7.77	21.1	14.4
**26 - D (application)**	BAC*	3760	992	4750	37.8	9.98	47.8	92.2	24.3	58.3
**27 - D (application)**	Phenothrin	26.9	55.4	82.3	37.8	77.6	115	92.1	189	141
**28 - D (application)**	Permethrin	182	245	427	26.4	35.6	62.0	64.3	86.9	75.6

* BAC = Benzalkonium chlorides

Table S5 Potential dermal exposure due to spraying and foaming of biocidal products – categorisation of results. The sample sizes n reflect different application scenarios but not necessarily individual operators.

**Analysed data sub-groups**	**Absolute exposure [µg]**<br>**Arithmetic mean ± standard deviation (median; range)**			**Normalised exposure [mg/kg]**<br>**Arithmetic mean ± standard deviation (median; range)**		
	**Coverall**	**Gloves**	**Total**	**Coverall**	**Gloves**	**Total**
**All devices (application only, n=24)**	2480 ± 4680 (370; 1.78–15700)	3440 ± 13900 (210; 0.295–68700)	5920 ± 17400 (639; 56.5–84400)	196 ± 291 (57.3; 2.16–1070)	101 ± 139 (23.3; 0.192–449)	297 ± 374 (119; 10.0–1280)
**Handheld devices (≤ 3 bar, small-scale applications) (application and wiping, n=6)**	646 ± 717 (327; 57.5–2020)	19300 ± 17600 (12600; 5550–52800)	20000 ± 18200 (13200; 5870–54800)	2660 ± 2060 (3100; 64.2–5400)	59500 ± 34000 (64800; 15100–102000)	62100 ± 35800 (67900; 15400–105000)
**Handheld devices (≤ 3 bar, small-scale applications) (application only, n=4)**	94.4 ± 75.6 (69.8; 33.2–205)	26.6 ± 20.7 (25.0; 7.62–48.7)	121 ± 66.9 (98.0; 72.9–215)	458 ± 153 (412; 329–679)	228 ± 225 (222; 19.9–449)	686 ± 323 (602; 414–1129)
**Propellant gas-assisted devices (n=5)**	237 ± 242 (147; 1.78–600)	159 ± 168 (82.3; 0.295–427)	396 ± 248 (428; 81.6–683)	416 ± 501 (146; 2.16–1067)	130 ± 106 (115; 0.529–258)	546 ± 540 (261; 83.3–1280)
**Low- and high-pressure devices (1–6 bar and >10 bar, large-scale applications) (n=15)**	3860 ± 5520 (1000; 34.2–15700)	5450 ± 17500 (330; 12.4–68700)	9300 ± 21500 (1770; 56.5–84400)	52.5 ± 56.5 (27.4; 3.34–200)	57.1 ± 104 (12.5; 0.192–364)	110 ± 145 (44.1; 10.0–448)
**Spray application (application only, n=9)**	3220 ± 5550 (383; 34.2–14800)	753 ± 1570 (71.8; 7.62–4750)	3970 ± 7030 (649; 56.5–19600)	275 ± 376 (81.5; 9.83–1070)	89.8 ± 150 (17.4; 0.192–449)	365 ± 500 (92.4; 10.0–1280)
**Foam application (application only, n=15)**	2030 ± 4220 (325; 1.78–15700)	5060 ± 17600 (297; 0.295–68700)	7080 ± 21500 (630; 72.9–84400)	148 ± 227 (48.6; 2.16–842)	107 ± 137 (26.7; 0.529–394)	256 ± 287 (147; 14.5–957)

Supplementary material – Figures

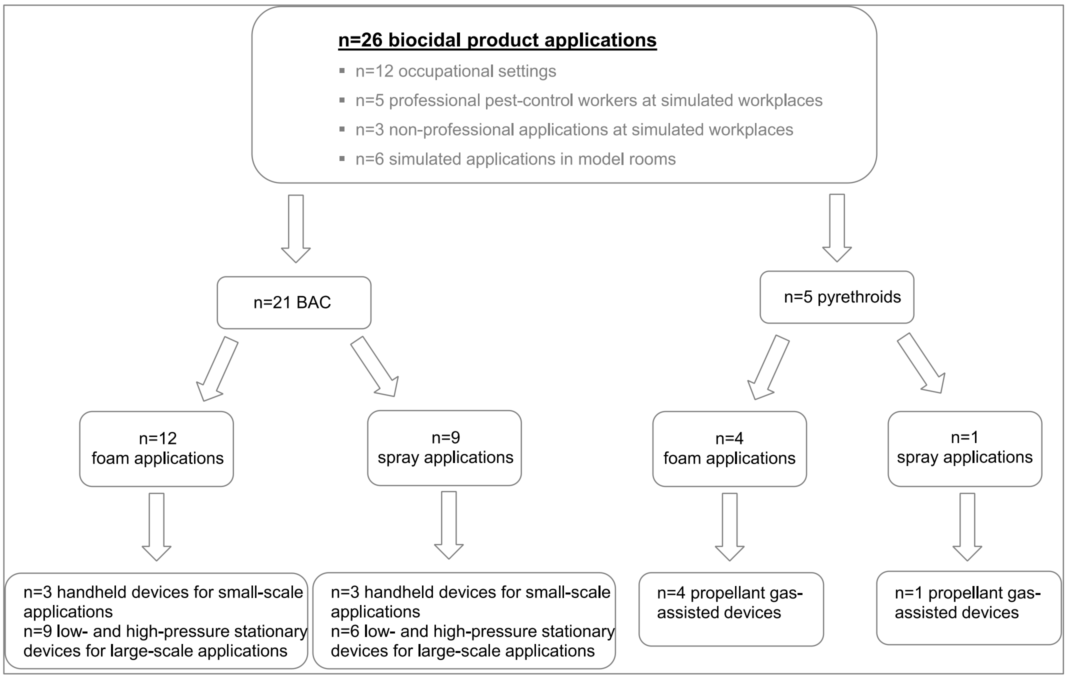

Figure S1 Schematic overview of exposure measurements.

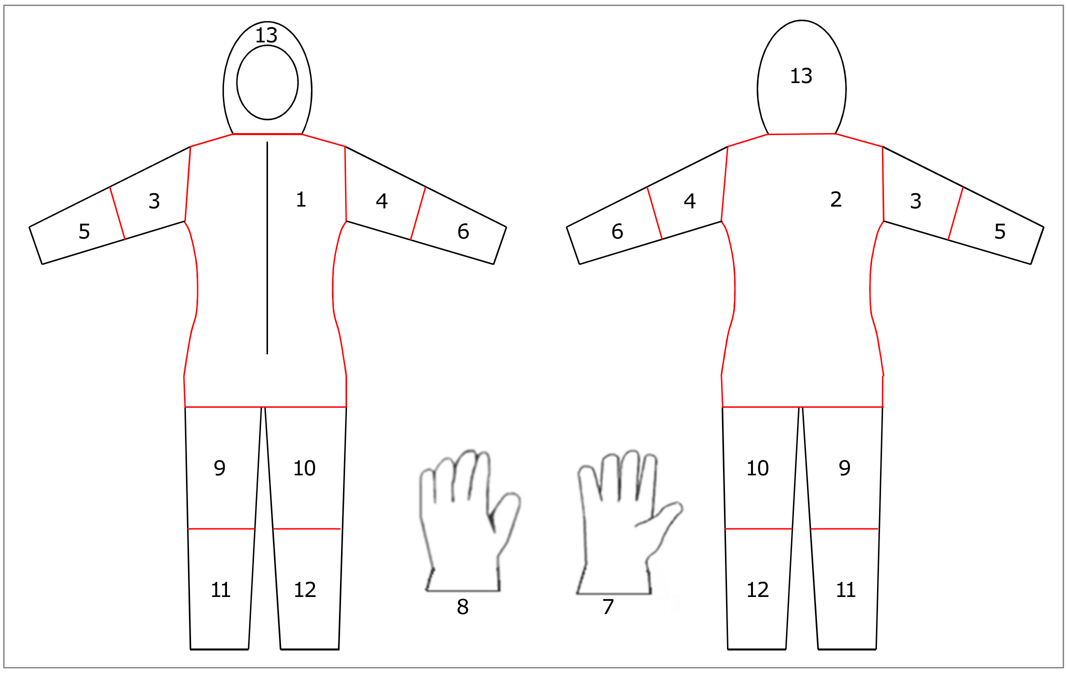

Figure S2 Schematic of sample segments for one sampling event generated from each sampling coverall and pair of gloves. Cutting guidance is indicated in red.

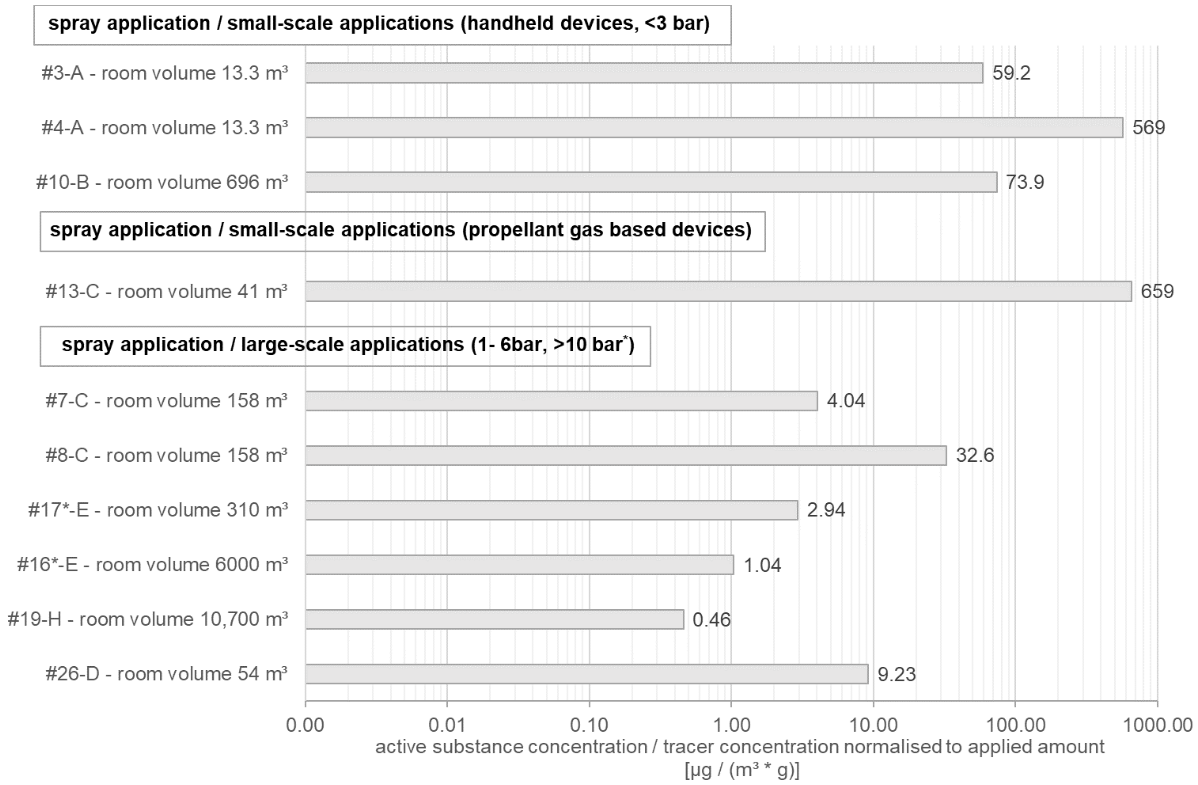

Figure S3 Spray application – inhalation exposure concentration normalised to applied amount of active substance / tracer. *High-pressure applications. Reservoir pressure of >10 bar.

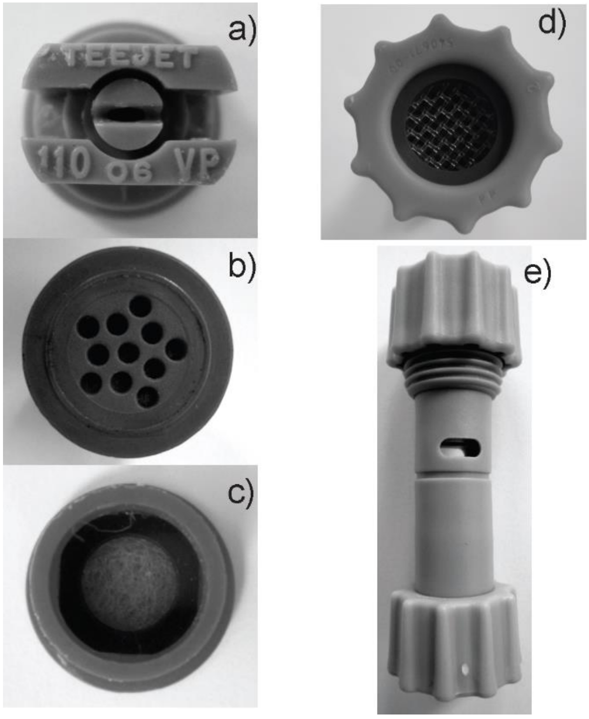

Figure S4 Foam nozzles for scenario #25 (a - c) and #24 (d and e). In scenario #25, foam was applied using a flat-fan nozzle (a) with the biocidal product-air mixture passing through a foam cartridge equipped with a felt washer (b and c) before reaching the nozzle. In scenario #24, a screen-based nozzle (d: outlet; e: sideways) was used.

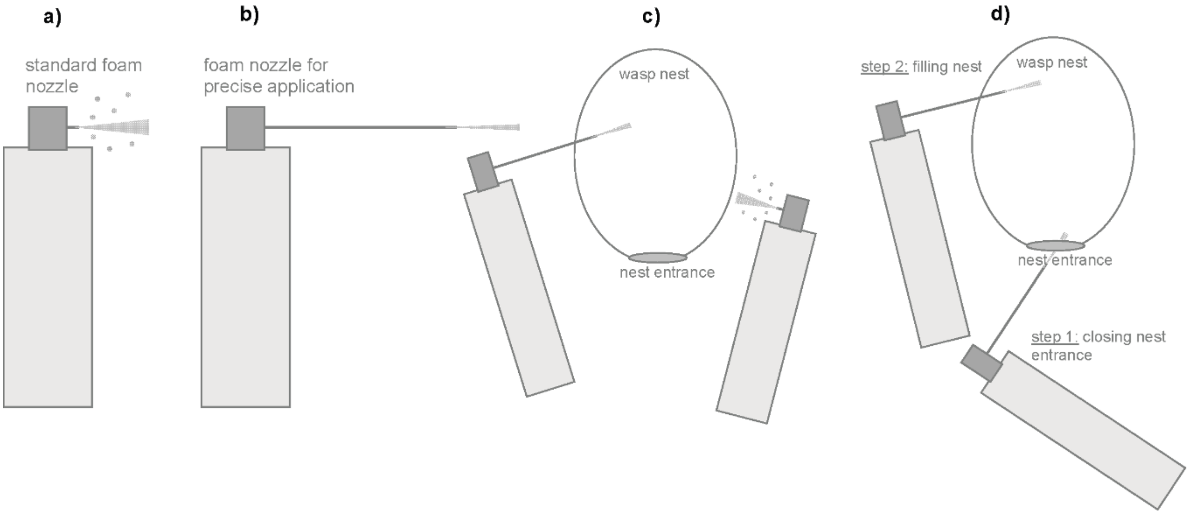

Figure S5 Schematic of propellant gas-based foam application – a) breaking-up of the foam jet observed for the standard nozzle; b) precise and exposure-reduced application with a designated foam nozzle; c) scenarios investigated in this study (as proposed by manufacturer); d) two-step treatment of a wasp nest as recommended by a pest-control professional.

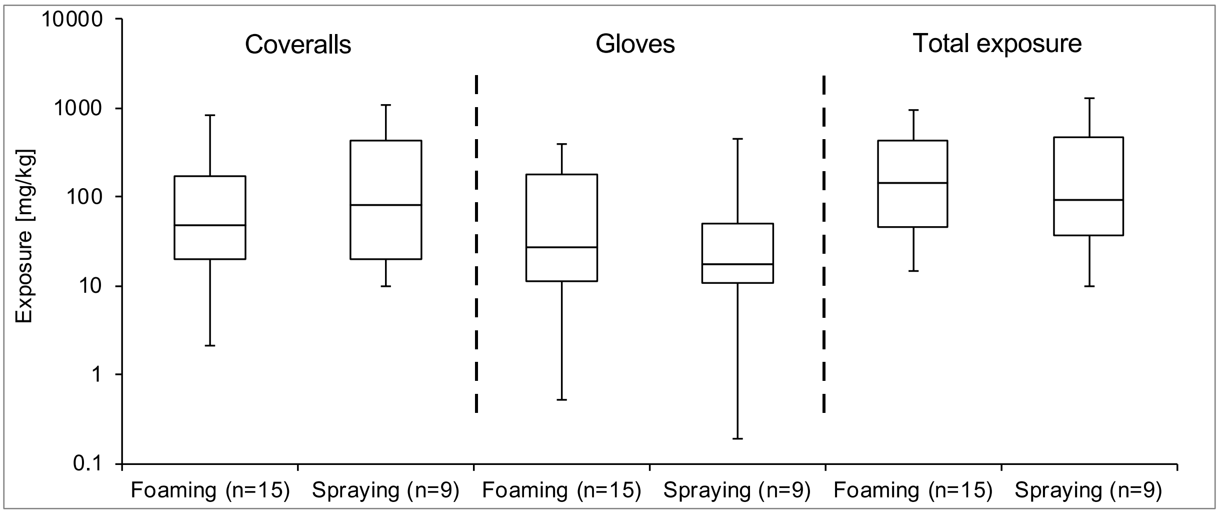

Figure S6: Dermal exposure on the coveralls and gloves (application only) for foam and spray applications, respectively. The box-plot visualisation reflects the exposure normalised to the applied amount of active substance [mg/kg], the sample size n different application scenarios but not necessarily individual operators.

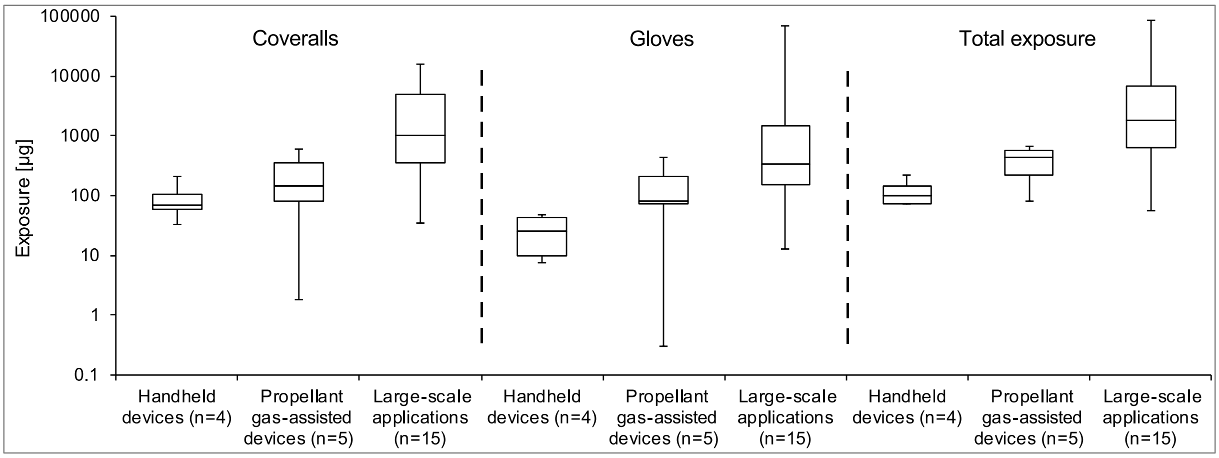

Figure S7 Dermal exposure on the coveralls and gloves (application only) categorised in three application-device groups. The box-plot visualisation reflects absolute exposure [µg], the sample size n different application scenarios in the respective category, but not necessarily individual operators.

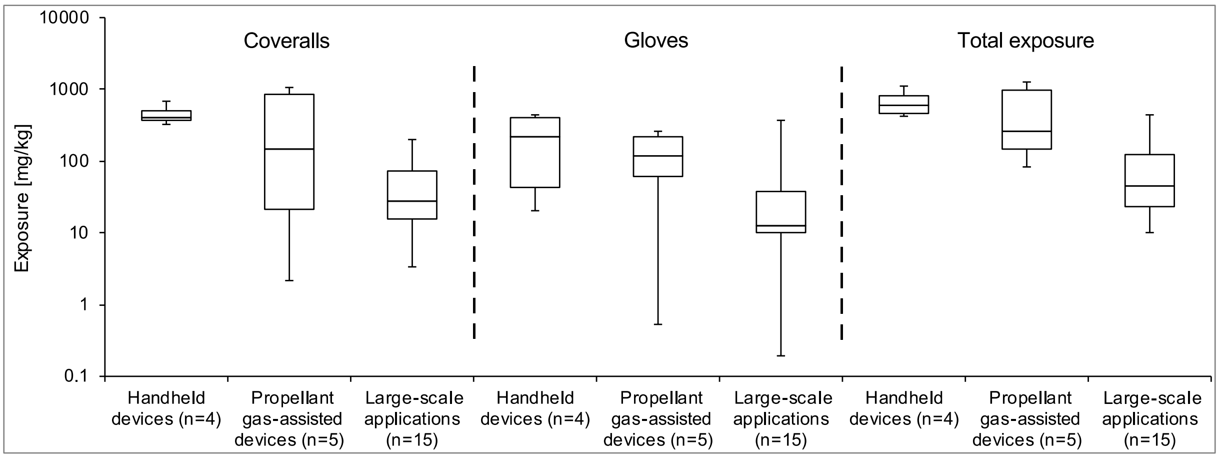

Figure S8 Dermal exposure on the coveralls and gloves (application only) categorised in three application-device groups. The box-plot visualisation reflects the absolute exposure normalised to the amount of active substance applied [mg/kg], the sample size n different application scenarios in the respective category, but not necessarily individual operators.

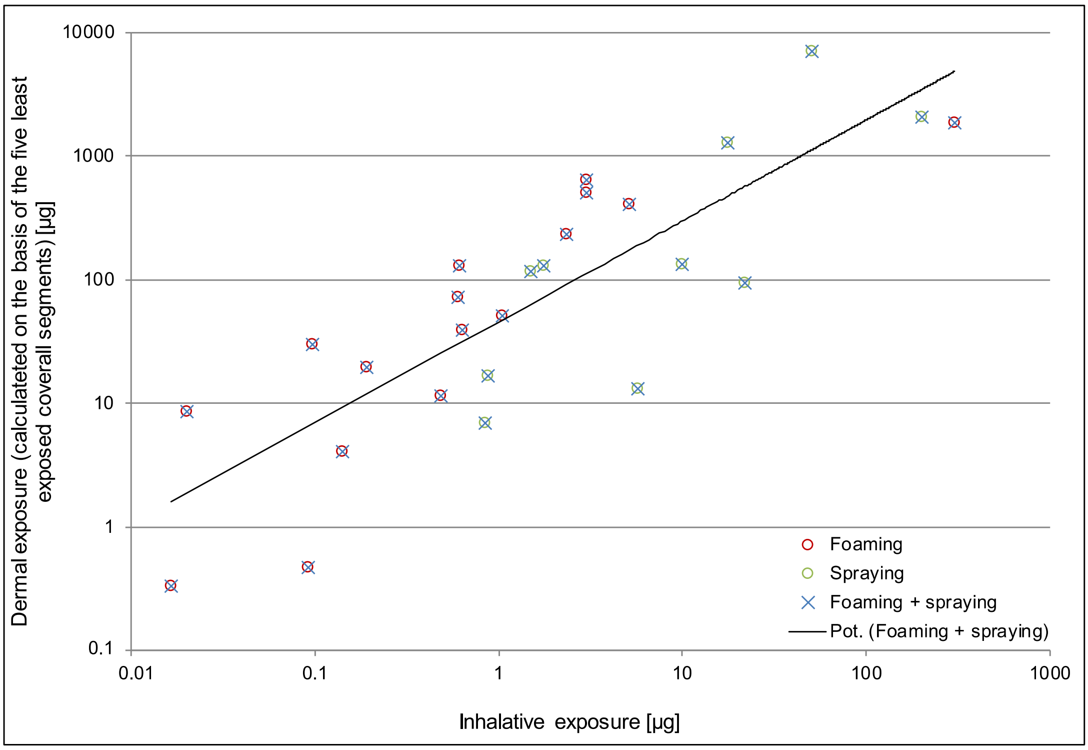

Figure S9 Log-log graph of the operators' dermal and inhalation exposure for foaming and spraying. For dermal exposure, "whole-body exposure by aerosol deposition" based on the five least-exposed coverall segments was used. The Pearson correlation was calculated using a two-tailed test (IBM SPSS Statistics; Pearson correlation coefficient r=0.830; p<0.01).

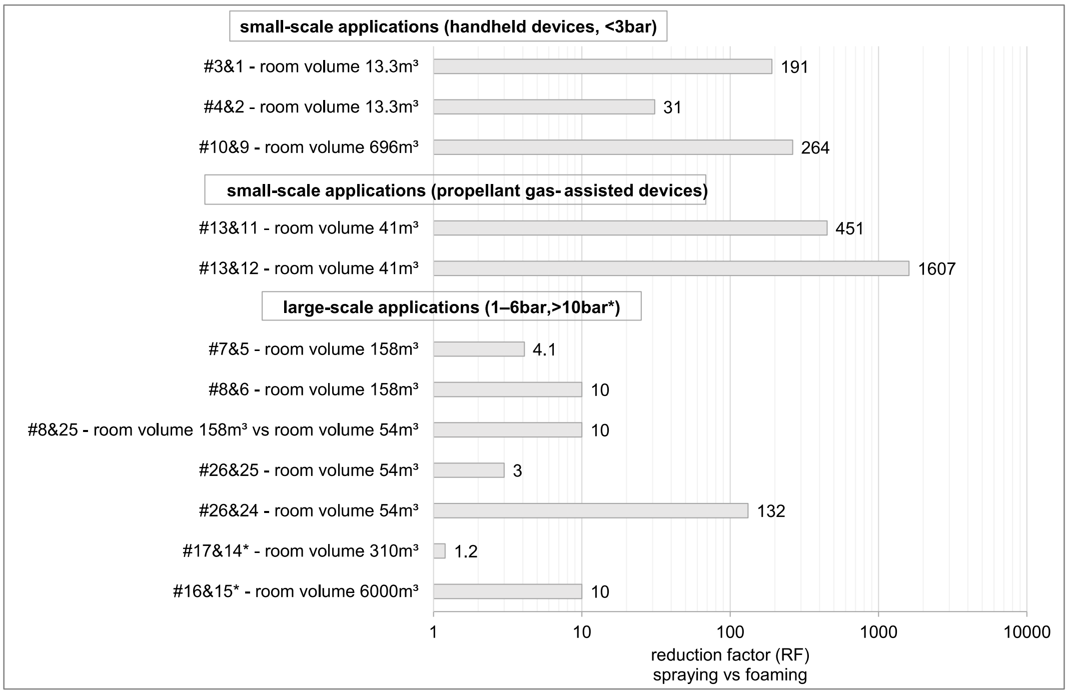

Figure S10 Inhalation exposure-reduction factor between purpose-driven spraying and foaming activities. Note: the exposure data for #14 and #15 were below the limit of quantification (LOQ). In those cases, the LOQ was used to calculate RF. *High-pressure applications. Reservoir pressure of >10 bar.
